# Remodeling the tumor immune microenvironment: mechanisms of crosstalk between regulated cell death macrophages

**DOI:** 10.3389/fimmu.2026.1933734

**Published:** 2026-08-27

**Authors:** Xiaotang Duan, Peiling Ma, Xinyue Ao, Xinyu Li, Jiaxin Huang, Linjiang Song

**Affiliations:** School of Medical and Life Sciences, Chengdu University of Traditional Chinese Medicine, Chengdu, China

**Keywords:** engineered nanoplatforms, immunotherapy resistance, regulated cell death, tumor immune microenvironment, tumor-associated macrophages

## Abstract

Although advances have been made in tumor immunotherapy, the immunosuppressive tumor microenvironment and treatment resistance still limit durable clinical benefit. Rather than functioning exclusively as terminal cytotoxic phenomena, regulated cell death (RCD) pathways generate surface cues, soluble mediators and cellular cargo that reshape immune responses. Here, we review the reciprocal interactions linking tumor-associated macrophages (TAMs) with apoptosis, necroptosis, pyroptosis, autophagy-related regulation, ferroptosis and cuproptosis. We examine how death execution, the kinetics and magnitude of tumor cell death, membrane integrity, and signal accessibility shape RCD-derived signals, and how macrophage lineage, functional state and signal-processing capacity determine their inflammatory and antitumor consequences. These reciprocal interactions regulate corpse clearance, antigen presentation, metabolic adaptation, tumor progression, and therapeutic resistance, while macrophages can in turn alter the susceptibility of viable tumor cells to death. We further discuss crosstalk among RCD pathways, extracellular vesicle-mediated communication, microbiota-related regulation and spatiotemporal reconfiguration during tumor progression and treatment. Finally, we evaluate nanomedicine strategies that coordinate tumor-selective RCD with macrophage regulation while emphasizing limitations in cell-type specificity, safety, patient selection and clinical evidence. This framework may guide more precise intervention along the RCD–macrophage axis.

## 1 Introduction

Cancer remains a major global health burden, with nearly 20 million new cases and 9.7 million deaths estimated in 2022 (1). During tumor evolution, malignant cells acquire functional capabilities that support survival, including resistance to cell death (2). Although surgery, radiotherapy, systemic therapy and immunotherapy have improved cancer control (3, 4), treatment resistance, residual disease and immune-refractory tumor states continue to limit durable clinical benefit (5). Many anticancer treatments directly or indirectly aim to eliminate malignant cells. However, dying cells are not passive endpoints. They expose or release signals and cellular material that can reshape the tumor microenvironment (TME) and influence subsequent immune and therapeutic responses (6). Therefore, the effect of tumor cell death depends not only on the extent of tumor cell elimination but also on how death-associated signals are sensed and processed within the TME.

Regulated cell death (RCD) comprises molecularly controlled processes that can be modified by genetic or pharmacological intervention (7, 8). This review focuses on apoptosis, necroptosis, pyroptosis, autophagy-related regulation, ferroptosis and cuproptosis. Autophagy usually supports cellular adaptation and recycling; autophagy-dependent cell death (ADCD) should be assigned only when the autophagic machinery is required for death (9, 10). The durable activity of venetoclax-based therapy in chronic lymphocytic leukemia (CLL) supports the clinical value of restoring apoptotic susceptibility, although comparable evidence remains limited for most nonapoptotic RCD pathways (11–13). Different death programs involve distinct execution mechanisms, metabolic requirements, membrane changes and signal outputs (14, 15). Dying cells expose or release combinations of surface cues, damage-associated molecular patterns (DAMPs), proteins, nucleic acids, oxidized lipids, metabolites and cellular material (16). These components are referred to here as an RCD-derived signal package. Its composition and extracellular availability vary with the identity and execution state of the dying cell, the timing of membrane damage and the efficiency of corpse clearance (17–19). Together, these factors determine which death-associated signals become available to immune cells and how long they persist (20). The key question is not only which death pathway is activated but also which signals become available and how they are interpreted.

Macrophages are major recipients of RCD-derived signals because they recognize and remove dying cells and process engulfed material (21, 22). However, tumor-associated macrophages (TAMs) are not a uniform population. Their developmental origin, functional state and tissue location shape their phagocytic, inflammatory, antigen-processing and immunoregulatory functions (23–25). Macrophages also act in the reverse direction by altering the susceptibility of viable tumor cells to subsequent RCD through direct and paracrine interactions (26). Accordingly, interactions between RCD and macrophages are shaped by determinants at three levels. On the death side, these include the identity and execution state of the dying cell, membrane integrity, and the kinetics and magnitude of tumor cell death. At the signal interface, the composition, accessibility and persistence of death-associated signals determine what macrophages encounter. On the macrophage side, cellular origin, functional state, receptor repertoire, phagocytic competence, intracellular processing and spatial location shape how these signals are interpreted. Together, these determinants shape macrophage polarization and functional-state transitions, thereby influencing inflammation, antigen processing and presentation, and antitumor immunity.

Previous reviews have described individual RCD pathways, immunogenic cell death (ICD), TAM regulation and RCD-based therapeutic strategies (26, 27). Here, we integrate these areas by comparing six RCD programs within a common framework that links death execution and signal generation to macrophage sensing and processing, while also considering how macrophages regulate the susceptibility of viable tumor cells to RCD. We further examine multipathway crosstalk, communication mediated by extracellular vesicles (EVs), microbiota-related regulation and spatial and temporal changes during tumor progression and treatment. We also evaluate nanomedicine strategies with respect to tumor selectivity, macrophage-targeting specificity, safety, patient selection and clinical feasibility. Throughout the review, we distinguish pathway activation from completed death, association from causality, and preclinical findings from clinical evidence. By centering on macrophage decoding, this review aims to examine how the molecular features, timing and magnitude of tumor cell death shape macrophage function, inflammation and therapeutic response.

## 2 Macrophage decoding of cell death signals

**Figure 1:**
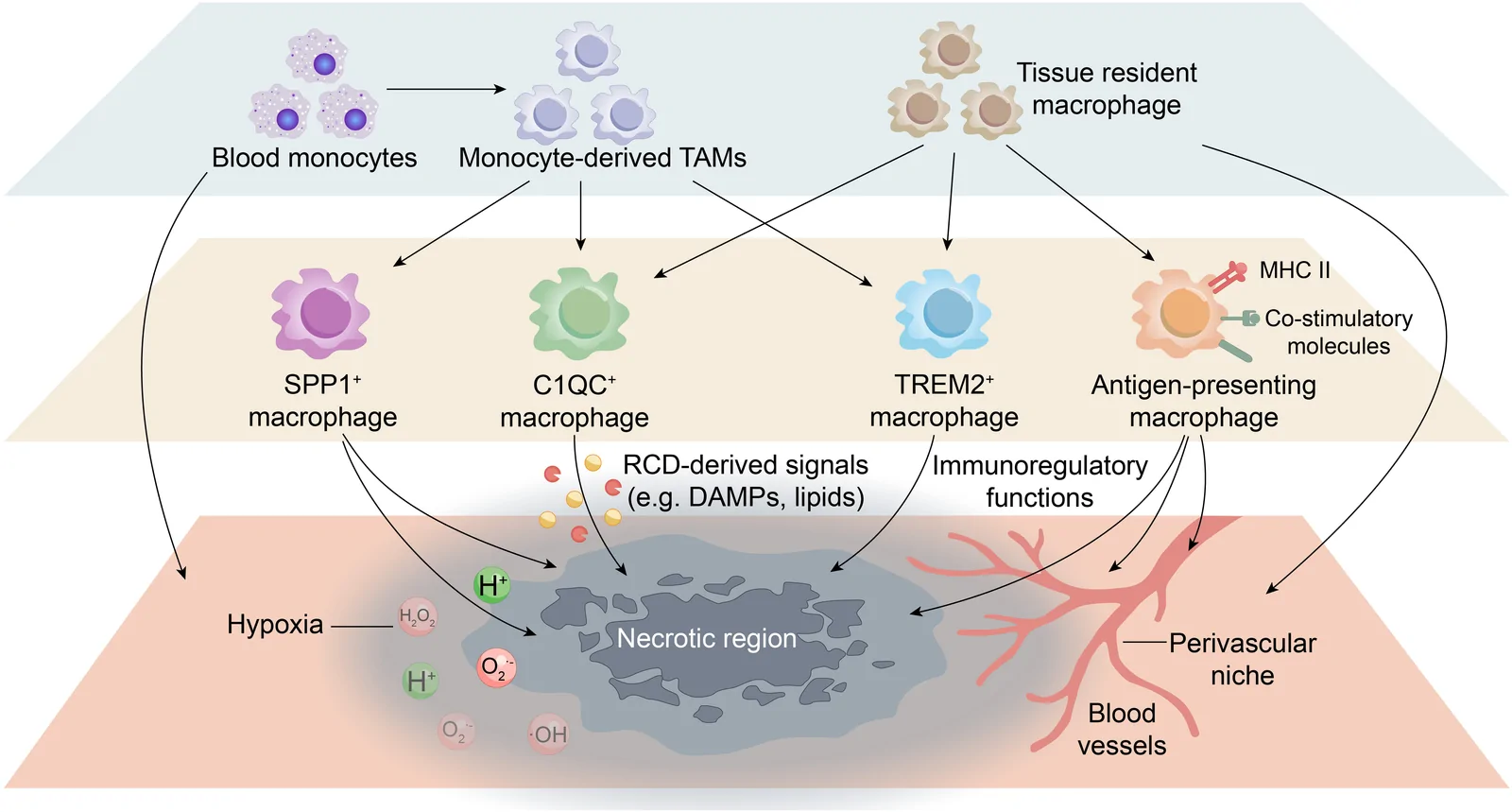
Macrophage heterogeneity determines the interpretation of RCD-derived signals. Tumor-associated macrophages comprise diverse populations shaped by cellular origin, developmental trajectory, spatial localization and metabolic state. Monocyte-derived macrophages and tissue-resident macrophages occupy distinct tumor niches, including hypoxic regions, necrotic areas, invasive fronts and perivascular compartments. These macrophage subsets exhibit specialized transcriptional and functional programmes, such as SPP1-positive, C1QC-positive and TREM2-associated states, which influence how extracellular signals generated by tumor-cell death are processed. Therefore, identical RCD-associated signals may elicit distinct macrophage responses depending on the receptor repertoire, intracellular metabolic state and local micro-environmental context. This heterogeneity provides a mechanistic basis for divergent outcomes of RCD–macrophage communication, ranging from immune activation to tumor-supportive inflammation.

TAMs comprise heterogeneous populations that differ in developmental origin, molecular state and spatial distribution. Single-cell and spatial studies have revealed recurrent TAM programs, but their abundance, localization and associated functions vary across tumor types and tissue regions (28–30). This heterogeneity shapes how macrophages encounter and process death-associated signals. The resulting response depends on the signals made accessible during death execution, their persistence and local abundance, and the sensing and processing capacity of the recipient macrophage (31). These variables link macrophage identity, death-signal decoding, and the timing and magnitude of tumor cell death to inflammatory and antitumor responses (**Figure 1**).

### 2.1 Macrophage heterogeneity in tumors

Macrophage heterogeneity in tumors reflects three related but nonequivalent dimensions: developmental origin, functional state and spatial position. The conventional M1–M2 model remains useful for describing responses to defined experimental stimuli, but it does not represent the range of macrophage states present in human tumors. Standardized descriptions of macrophage activation should specify the cellular source, activating stimulus and measured markers rather than assume that all cells belong to one of two fixed populations (32, 33). Single-cell studies have identified recurrent TAM programs, yet a unified nomenclature and a stable correspondence between individual markers and functions are lacking (34, 35). Tissue dissociation, incomplete cell recovery, transcriptional activation during sample processing and erroneous assignment of macrophage transcripts can further distort the apparent boundaries between subpopulations (36). Accordingly, the terms M1-like and M2-like should be retained only when they reflect the stimuli, markers or phenotypes measured in the original study. In other settings, macrophages are better described by their observed functions, including phagocytosis, cytokine production, antigen presentation, metabolic activity and regulation of lymphocyte responses.

Developmental origin provides the first layer of TAM heterogeneity. Tumor macrophages can be derived from tissue-resident populations established during development or from circulating monocytes recruited from the blood. Tissue-resident macrophages have organ-specific developmental trajectories, transcriptional programs and life cycles that contribute to their baseline tissue functions (37). In early non-small cell lung cancer (NSCLC), tissue-resident macrophages accumulate near tumor cells, promote tumor cell invasion and support regulatory T-cell responses. As tumors progress, these macrophages move toward the lesion periphery, whereas monocyte-derived macrophages become dominant within the tumor (38). These findings reveal that macrophage composition changes with disease stage and suggest that ontogeny provides a developmental starting point rather than a fixed tumor-associated function because local tissue conditions can further reshape both resident and recruited macrophages.

Functional state provides a second and more dynamic layer of heterogeneity. Single-cell analyses frequently identify TAM programs enriched for secreted phosphoprotein 1 (SPP1), complement C1q C chain (C1QC) or triggering receptor expressed on myeloid cells 2 (TREM2), but these markers do not define mutually exclusive lineages or universal functions (39). SPP1 and TREM2 were coexpressed by a TAM population enriched in human and murine metastatic prostate tumors, demonstrating that marker-defined programs can overlap within the same cells (40). The functional interpretation is also tissue dependent. In malignant pleural effusion, complement component 1q-positive (C1q^+^) TAMs displayed increased fatty acid metabolism, expressed inhibitory molecules and impaired CD8^+^ T-cell activity, but these findings do not establish an equivalent function for every C1QC-enriched TAM population (41). NLR family pyrin domain containing 3 (NLRP3) provides a further example of context dependence. In a subcutaneous lung cancer model, TREM2 deletion restricted tumor growth and enhanced CD4^+^ T-cell and natural killer (NK) cell activity, whereas in a spontaneous pancreatic ductal adenocarcinoma (PDAC) model, TREM2 depletion increased NLRP3–interleukin-1β (IL-1β)-dependent pathogenic inflammation and accelerated tumor progression (42, 43). These results identify inflammasome activity and the surrounding tissue-specific immune network as key determinants of the response to triggering receptor expressed on myeloid cells 2 perturbation. Marker-defined TAM programs are therefore most informative when interpreted together with their location, pathway activity and measured function.

Spatial heterogeneity provides a third layer by shaping which cells, metabolites and death-associated material are accessible to each macrophage. In human breast and colon tissues, SPP1^+^ macrophages were enriched in hypoxic and necrotic tumor regions and were associated with poor outcomes in patients with colon cancer, whereas interleukin 4 induced 1-positive (IL4I1^+^) macrophages occupied regions of high cell turnover and engulfed dying cells (44). In a spontaneous mammary tumor model, lymphatic vessel endothelial hyaluronan receptor 1-positive (LYVE1^+^) TAMs localized near blood vessels and supported the expansion of perivascular mesenchymal cells, forming a proangiogenic niche (45). Spatial separation within mammary tumors also resulted in the generation of TAM populations with distinct transcriptional programs and different capacities to phagocytose tumor cells and activate CD8^+^ T cells (46). In diffuse glioma, monocyte-derived TAMs in perinecrotic regions acquired a hypoxia-associated program that increased vascular permeability and impaired drug delivery (47). Thus, ontogeny, functional state and spatial position describe different aspects of macrophage identity. Together, these factors define the context in which macrophages encounter, internalize and process RCD-derived signals.

### 2.2 Recognition and processing of death-associated signals

Dying cells do not provide macrophages with isolated signals. They expose or release combinations of surface cues, soluble mediators, proteins, lipids, nucleic acids, metabolites and cellular material. Together, these components constitute an RCD-derived signal package. The immune activity of these signals depends on their molecular identity, extracellular availability and persistence (31, 48). Productive adaptive immunity further requires sufficient antigenicity and adjuvanticity within a permissive microenvironment (49, 50). Here, we organize macrophage decoding around three variables: signal accessibility, signal persistence, and the sensing and processing pathways present in the recipient cell.

Macrophage decoding begins with signal accessibility and recognition. Surface-associated cues support contact with and engulfment of dying cells, whereas exposed or released DAMPs activate receptors on the plasma membrane or within intracellular compartments. Efferocytosis enables phagocytes to recognize, internalize and digest apoptotic cells and induces phenotypic changes that promote inflammation resolution (51, 52). Recognition alone does not define engulfment capacity. Increased plasma membrane abundance, together with reduced membrane tension, enhanced macrophage uptake of apoptotic corpses and cancer cells. These findings identify membrane availability as a biophysical constraint on phagocytic capacity (53). Plasma membrane integrity influences whether intracellular components remain contained or become available to neighboring cells, while the timing of membrane permeabilization changes the sequence in which signals are encountered. Thus, different RCD modalities generate distinct patterns of signal exposure even when they release some of the same molecules (54, 55). The cellular source and location of DAMPs further influence whether sensing occurs within the affected cell, in neighboring cells, including macrophages, or across more distant tissues (56). Corpse clearance is consequently part of the decoding process because it limits signal persistence, transfers cellular cargo into phagolysosomal compartments and may prevent the secondary release of intracellular material (57).

The state of the recipient macrophage further shapes how an accessible signal package is processed. TAM populations differ in their phagocytic capacity, receptor expression, metabolic adaptation and interactions with lymphocytes and stromal cells. These properties influence whether material from dying cells is cleared, inflammatory mediators are produced, tumor antigens are processed or tissue-remodeling and immunoregulatory programs are engaged (58). Engulfment itself can reshape macrophage function. In syngeneic and autochthonous lung tumor models, phagocytic TAMs showed increased oxidative phosphorylation (OXPHOS) and greater expression of antigen-presentation and anti-inflammatory proteins, together with reduced classical proinflammatory effectors (59). These findings indicate that the uptake history of a macrophage can alter its metabolic and functional state. Inflammatory gene expression alone should not be equated with antitumor activity, and functional conclusions should be based on direct measurements of corpse uptake, cytokine production, antigen processing, lymphocyte regulation or tumor outcome (60).

Evidence for macrophage decoding should also distinguish association from causality. Activation of a proximal death pathway does not necessarily lead to completed cell death (61, 62). For example, sublethal mitochondrial outer-membrane permeabilization (MOMP) and cytochrome c release can occur without apoptotic execution and can instead generate drug-tolerant persister cells (63). Therefore, RCD-related gene signatures or pathway activation alone should be treated as evidence of pathway engagement rather than proof of irreversible death. Marker-defined macrophage clusters likewise establish transcriptional associations unless lineage, localization and function are supported by additional experiments. DAMP release and local inflammation are also insufficient to establish ICD, which requires dying cells to induce an adaptive immune response associated with immunological memory (64). The strongest evidence for an RCD–macrophage interaction combines confirmation of the death modality, perturbation of the proposed signal or recognition pathway and measurement of a macrophage function linked to tumor outcome. A mechanistic comparison of RCD–macrophage interactions should therefore establish a causal sequence from completed death in a defined source cell to an accessible signal or cargo, its recognition and processing by a specified macrophage population, and a measurable macrophage function that influences tumor behavior or treatment response.

### 2.3 Timing and magnitude of tumor cell death

At the population level, cell death kinetics can be described by the time to death onset and the maximal death rate, whereas death magnitude can be summarized by the lethal fraction measured at a defined time (65, 66). Similar endpoint responses can arise from different combinations of proliferation and death rates (67). Thus, the same final lethal fraction can result from rapid or gradual killing. These metrics capture distinct features of the dying-cell population. In this review, we interpret their immune relevance through the timing and amount of material presented to macrophages. The same lethal fraction can therefore correspond to different temporal patterns of macrophage exposure when cell killing proceeds at different rates, while the amount of material that persists locally also reflects corpse clearance.

In a 4T1 mammary tumor model, pyroptosis of fewer than 15% of tumor cells was sufficient to eliminate the entire tumor graft, and tumor regression required an intact immune system and T cells (68). In a ferroptosis vaccination model, early ferroptotic cancer cells promoted dendritic cell maturation and protective antitumor immunity, whereas late ferroptotic cells did not (69). Together, these observations indicate that endpoint cell loss alone does not capture the immune consequences of tumor cell death. Neither study was designed to define a macrophage dose response to death rate or lethal fraction.

Macrophage responses are further shaped by how dying-cell material is cleared and processed. In breast cancer models, efferocytosis of chemotherapy-induced apoptotic cells activated TANK-binding kinase 1 (TBK1), increased interleukin-10 (IL-10) production and promoted an M2-like TAM state. Thymosin α-1 activated SH2-containing inositol 5′-phosphatase 1 (SHIP1) through lysosomal Toll-like receptor 7 (TLR7)–myeloid differentiation primary response 88 (MyD88) signaling, reduced TBK1 activity and macrophage IL-10 production, and improved epirubicin efficacy (70). Efferocytosis also activated NLRP3-dependent IL-1β secretion in tumor-infiltrating myeloid cells, promoting tumor growth without requiring gasdermin D (GSDMD)-dependent pyroptosis (71). In colorectal cancer models, small interfering RNA (siRNA)-mediated MER proto-oncogene tyrosine kinase (MERTK) knockdown in TAMs reduced efferocytosis, increased apoptotic cell accumulation and intratumoral immune activation, suppressed tumor growth, and enhanced programmed cell death protein 1 (PD-1) blockade (72).

Together, these studies show that the uptake and processing of dying-cell material can alter macrophage polarization, inflammatory output and treatment response. In the framework used here, death kinetics and magnitude define the temporal and quantitative input, whereas clearance and post-engulfment processing determine how that input is handled. However, the relationship between tumor cell death rate or lethal fraction and these macrophage outcomes has not been quantified. Future studies should pair time-resolved measurements of death onset, death rate and lethal fraction with corpse burden, clearance time, cytokine production, antigen processing, lymphocyte regulation and tumor response. Such analyses could distinguish graded dose responses from threshold effects and discrete temporal windows.

## 3 Regulated cell death and macrophages

### 3.1 Apoptosis

#### 3.1.1 Signal exposure during apoptosis

Apoptosis is an RCD program that is typically executed by caspases and initiated through death-receptor or mitochondrial pathways (73, 74). Death-receptor signaling activates caspase-8, whereas BCL-2-associated X protein (BAX)- and BCL-2 antagonist/killer 1 (BAK1)-mediated MOMP releases cytochrome c and enables apoptosome-dependent activation of caspase-9; both pathways converge on caspase-3 and caspase-7 (75–78). During early execution, plasma membrane integrity is generally retained, although dying cells shrink, undergo membrane blebbing and may fragment into membrane-bound apoptotic bodies (79–81). Apoptotic cells externalize phosphatidylserine (PS), which provides a major cue for phagocytic recognition (82), and selectively release nucleotides, lipids and metabolites through regulated routes (83–86). Pannexin 1 (PANX1) channels contribute to the release of a defined subset of this apoptotic metabolite secretome (87, 88). During this membrane-intact phase, macrophages encounter surface PS, regulated soluble signals, and membrane-enclosed cargo. Outside tumor models, apoptotic-cell fragments and the composition of engulfed apoptotic-cell cargo can activate and transcriptionally reprogram macrophages (89, 90). The duration of the membrane-intact phase therefore provides a temporal window during which surface cues, regulated soluble signals, and membrane-enclosed cargo remain accessible before corpse clearance or loss of membrane integrity.

#### 3.1.2 Efferocytosis and control of tumor apoptosis

Efferocytosis begins with the recognition and uptake of apoptotic cells, while receptor signaling and post-engulfment processing provide distinct control points (91). T-cell immunoglobulin and mucin domain-containing protein 4 (TIM-4) directly captures PS-bearing cells, whereas growth arrest-specific protein 6 (GAS6) bridges PS to MERTK to promote internalization (92, 93). MERTK signaling coordinates phagocytic-cup closure through calcium-dependent filamentous actin (F-actin) disassembly and myosin-II contraction (94). After uptake, phagolysosomal degradation converts apoptotic cell constituents into metabolic signals (95). In nontumor resolution models, corpse-derived methionine supports DNA methyltransferase 3 alpha (DNMT3A)-dependent proresolving signaling, whereas arginine conversion to putrescine promotes Rac family small GTPase 1 (RAC1)-dependent cytoskeletal remodeling and serial efferocytosis (96, 97). Whether these cargo-derived programs operate across TAM subsets remains unresolved. In CT26 peritoneal tumor and malignant ascites models, apoptotic-cell-derived PS activated focal adhesion kinase (FAK)–SRC proto-oncogene, nonreceptor tyrosine kinase (SRC)–signal transducer and activator of transcription 3 (STAT3) signaling, increased lysine demethylase 6B (KDM6B; also known as JMJD3), arginase 1 (ARG1) and mannose receptor C-type 1 (MRC1; also known as CD206) expression in macrophages, and promoted tumor growth (98). These findings identify uptake and post-engulfment metabolism as separate control points. In a murine melanoma model, efferocytosis also induced proangiogenic macrophage function and remodeled chromatin accessibility, attenuating macrophage responsiveness to interferon-γ (99).

Macrophages also alter the apoptotic thresholds of neighboring tumor cells. In colorectal cancer, increased infiltration by sialoadhesin-positive (CD169^+^) macrophages was associated with improved overall survival. Macrophage depletion, coculture and receptor-blocking experiments further revealed that CD169–CD43 engagement enabled contact-dependent Fas ligand (FasL)–Fas cell surface death receptor (Fas) signaling and tumor cell apoptosis (100). This represents a receptor-defined macrophage effector function rather than a generic polarization state. Conversely, in esophageal squamous cell carcinoma, TAM-derived C-C motif chemokine ligand 22 (CCL22) activated diacylglycerol kinase-α (DGKα) in tumor cells. DGKα signaling limited reduced nicotinamide adenine dinucleotide phosphate (NADPH) oxidase 4 (NOX4)-dependent reactive oxygen species (ROS) accumulation and increased nuclear factor kappa B (NF-κB)-dependent adenosine triphosphate (ATP)-binding cassette transporter expression, thereby reducing the level of intracellular cisplatin and suppressing apoptosis (101). These mechanisms act at a different stage from corpse processing because they determine whether viable tumor cells enter apoptosis. Macrophage control of the apoptotic threshold can therefore change the amount of apoptotic material generated after treatment and the subsequent clearance demand.

#### 3.1.3 Apoptotic clearance and treatment response

The functional consequences of apoptotic cell uptake depend on the recipient macrophage population and the intracellular fate of the engulfed material. In prostate tumor models in bone, macrophage efferocytosis of apoptotic tumor cells activated STAT3 and nuclear factor-κB signaling and generated a C-X-C motif chemokine ligand 5 (CXCL5)-dependent inflammatory response that accelerated tumor growth in bone (102). In contrast, during early ovarian tumor seeding, TIM-4^+^ large resident peritoneal macrophages captured PS-bearing tumor material and formed phagosomes with delayed acidification and cargo degradation. This processing route preserved tumor antigens for cross-presentation, whereas *Tim4* deletion reduced tumor-specific effector CD8^+^ T-cell responses and accelerated tumor growth in mice (92). Together, these models illustrate that macrophage identity and phagosomal processing can shape whether apoptotic cargo drives tumor-supporting inflammation or is retained for cross-presentation.

Apoptosis–macrophage interactions also contribute to therapeutic resistance through effects on viable tumor cells and treatment-generated cellular material. Tumor–macrophage signaling circuits can increase the apoptotic threshold of malignant cells, suppress treatment-induced oxidative stress or increase drug efflux, thereby contributing to resistance to chemotherapy, including resistance to 5-fluorouracil and cisplatin (101, 103, 104). Treatment can also generate a concentrated burden of apoptotic material. For apoptosis, death magnitude and clearance kinetics jointly shape the local corpse burden. The amount of apoptotic material generated and the rate at which it is removed determine how long this material remains available for macrophage processing. In human epidermal growth factor receptor 2 (HER2)-positive mouse mammary tumor virus (MMTV)-Neu transgenic mice, MERTK-dependent efferocytosis cleared apoptotic tumor cells. Blocking efferocytosis promoted secondary necrosis, which increased indoleamine 2,3-dioxygenase 1 (IDO1) activity and sustained immunosuppressive features (105). In experimental treatment models, after phagocytosing therapy-generated tumor debris, medullary sinus macrophages in tumor-draining lymph nodes induced the expression of interleukin-33 (IL-33), thereby activating regulatory T cells (Tregs) and suppressing the function of intratumoral CD8^+^ T cells. Macrophage-specific *Il33* deletion or blockade of its receptor, interleukin 1 receptor-like 1 (IL1RL1; also known as ST2) improved responses to targeted therapy and PD-1 blockade in mice (106). These findings show that treatment-generated dying-cell material can trigger a macrophage-dependent phase of immune suppression after tumor cell killing.

Together, these studies support an apoptosis-specific conceptual burden–clearance model. Death magnitude determines the amount of apoptotic material generated, whereas clearance kinetics determines its persistence and processing by macrophages. The studies reviewed here do not define a quantitative threshold at which apoptotic-cell generation exceeds local clearance capacity, nor do they establish a generalizable quantitative relationship between the burden-to-clearance balance and macrophage state, inflammatory output, or therapeutic efficacy across tumor contexts.

### 3.2 Necroptosis

#### 3.2.1 Necroptotic signaling and membrane rupture

Necroptosis is a regulated lytic cell death program executed principally by receptor-interacting protein kinase 3 (RIPK3) and mixed lineage kinase domain-like pseudokinase (MLKL) (107). In tumor necrosis factor receptor 1 (TNFR1) signaling, the proteolytic activity of caspase-8 restrains receptor-interacting protein kinase 1 (RIPK1)–RIPK3 complex formation. When this restraint is lost, RIPK1 can recruit RIPK3 and support necrosome assembly (108–111). Activated RIPK3 phosphorylates MLKL, which oligomerizes and translocates predominantly to the plasma membrane, disrupting ionic homeostasis and promoting cell swelling and plasma membrane permeabilization (112–114). Terminal plasma membrane permeabilization and rupture permit the extracellular release or exposure of intracellular proteins, nucleotides and other cellular material (115). However, necroptotic pathway activation does not always cause rapid lysis. In hepatocytes with low RIPK3 expression, concurrent necrosome and nuclear factor-κB (NF-κB) signaling results in a prolonged sublethal state with limited membrane permeabilization and chemokine secretion rather than immediate death (116). Therefore, RIPK3 or MLKL activation, sublethal necroptotic signaling and completed necroptosis should be distinguished experimentally. Before terminal rupture, regulated chemokine production can recruit or activate myeloid cells. Membrane failure subsequently broadens macrophage exposure to intracellular proteins, nucleotides, and cellular material.

#### 3.2.2 Macrophage responses and control of necroptosis

Macrophages decode signals generated at different stages of necroptotic stress. Before terminal rupture, sublethal necrosome–NF-κB signaling in hepatocytes induces C-C motif chemokine ligand 20 (CCL20) and CCL2, promoting the accumulation of monocyte-derived macrophage populations associated with hepatocarcinogenesis (116). In PDAC, RIPK1–RIPK3 signaling generated parallel C-X-C motif chemokine ligand 1 (CXCL1)- and macrophage-inducible C-type lectin (MINCLE)-dependent networks that promoted macrophage-mediated adaptive immune suppression and tumor progression (117). Spliceosome-associated protein 130 (SAP130), a damaged-cell ligand recognized by MINCLE, linked released cellular material to myeloid-cell activation (118). These studies suggest that macrophage regulation can occur at different stages of necroptotic stress. Sublethal necrosome–NF-κB signaling can recruit monocyte-derived macrophages through chemokines before rapid lysis, whereas SAP130 released from damaged or dead cells can engage MINCLE once intracellular material becomes extracellularly accessible.

Macrophages can themselves execute necroptosis. In bone marrow-derived macrophages (BMDMs), the RIPK1 S161N substitution partially reduced RIPK1 catalytic activity, MLKL phosphorylation and necroptotic death, supporting a role for S161 autophosphorylation in RIPK1-dependent necroptosis (119). *In vitro* M1-polarizing conditions increased RIPK3, MLKL and Z-DNA-binding protein 1 (ZBP1) expression and increased the susceptibility of BMDMs to RIPK3-dependent lytic cell death compared with unstimulated or M2-polarized cells (120). These findings are restricted to defined experimental conditions and do not establish stable TAM lineages.

Macrophages can also alter the necroptotic thresholds of neighboring tumor cells. In colorectal cancer (CRC), M2-polarized TAMs increased methyltransferase-like 3 (METTL3)-dependent N6-methyladenosine (m6A) modification in tumor cells, with TNF receptor-associated factor 5 (TRAF5) identified as a downstream mediator linking this program to reduced necroptosis and oxaliplatin resistance (121). Whether tissue-resident and monocyte-derived TAM populations differ in their localization to necroptotic regions, sensing of released material, or intrinsic susceptibility to necroptosis remains unresolved.

#### 3.2.3 Cell-specific effects of necroptosis

The consequences of necroptosis depend on the cell type undergoing death and the subsequent macrophage response. In PDAC, MLKL-driven necroptosis increased tumor cell cluster of differentiation 47 (CD47) signaling and induced macrophage extracellular traps, which linked macrophage recruitment and impaired phagocytic control to liver metastasis (122). A separate PDAC study identified a feedback circuit in which TRAF3-interacting protein 2 antisense RNA 1 (TRAF3IP2-AS1) deficiency increased tumor cell necroptosis and the release of transforming growth factor-β1 (TGF-β1). TGF-β1 induced an M2-like immunosuppressive macrophage phenotype and further TGF-β1 production; accumulated TGF-β1 then increased TRAF3IP2-AS1 transcription in tumor cells and limited further necroptosis (123). A third PDAC model linked parallel CXCL1 and SAP130–MINCLE signaling to macrophage-mediated adaptive immune suppression (117, 124). Together, these models identify CD47-dependent phagocytic inhibition, macrophage extracellular traps, TGF-β1 feedback, and SAP130–MINCLE signaling as distinct routes linking tumor-cell necroptosis to immune suppression.

Treatment can create a distinct route of necroptosis–macrophage communication. In breast and ovarian cancer models, chemotherapy selectively activated poly(ADP-ribose) polymerase 1 (PARP1) in tumor endothelial cells, leading to poly(ADP-ribosyl)ation (PARylation) of MLKL and endothelial necroptosis. Endothelial C-X-C motif chemokine ligand 12 (CXCL12) signaling through C-X-C motif chemokine receptor 4 (CXCR4) suppressed the generation of CXCL10^+^ macrophages and disrupted their antitumor interaction with CXCR3^+^ CD8^+^ T cells, thereby promoting chemotherapy resistance (125). Together with the TAM–METTL3–TRAF5 pathway described above, resistance can arise because stromal necroptosis changes macrophage function or because macrophages suppress necroptosis in tumor cells (121). Conversely, a radio-activable gold-single-atom-based artificial enzyme (Au-RadioSAE) induced tumor cell necroptosis, increased CD8^+^ T-cell infiltration and promoted an M1-like macrophage phenotype in a CT26 mouse model, reducing tumor growth and recurrence (126). This study provides murine proof of principle rather than evidence of clinical efficacy.

Necroptotic effects on macrophages are governed mainly by execution stage and cellular source. Sublethal signaling can recruit myeloid cells before lysis, whereas membrane rupture broadens signal exposure, and tumor-cell and stromal necroptosis engage distinct immune circuits. How necroptotic burden quantitatively shapes macrophage function remains unclear, and transcriptomic associations alone cannot resolve this relationship (127–129).

### 3.3 Pyroptosis

#### 3.3.1 Gasdermin pores and signal release

Pyroptosis is a regulated lytic cell death program executed by pore-forming members of the gasdermin family (130). Proteolytic cleavage releases the cytotoxic gasdermin N-terminal domain from C-terminal autoinhibition, enabling membrane binding, oligomerization and pore formation (131). The resulting ionic imbalance causes cell swelling and increased plasma membrane permeability and may culminate in terminal rupture when membrane damage becomes irreversible (132). In the canonical pathway, pattern-recognition receptors assemble inflammasomes that activate caspase-1, leading to GSDMD cleavage and maturation of pro-interleukin-1β (pro-IL-1β) and pro-interleukin-18 (pro-IL-18) (133). In the noncanonical pathway, cytosolic lipopolysaccharide (LPS) directly activates caspase-4 and caspase-5 in humans or caspase-11 in mice, leading to GSDMD cleavage and secondary NLRP3 inflammasome activation (134, 135). Inflammasome-independent execution routes include caspase-3-mediated gasdermin E (GSDME) cleavage and granzyme A-mediated gasdermin B (GSDMB) cleavage (136, 137). These pathways release or expose cytokines, nucleotides, nucleic acids, proteins and other cellular material, with the composition and timing of signal availability determined by the executor gasdermin and the extent of membrane damage (138, 139). Because pore formation can precede terminal rupture, gasdermin cleavage, cytokine release, and terminal lysis represent distinct experimental endpoints. Pyroptosis-related expression or gene signatures alone do not establish completed cell death or immunogenic cell death.

#### 3.3.2 Macrophage responses and susceptibility to pyroptosis

Pyroptotic cells influence macrophages through cytokines and intracellular cargo, whereas macrophages can reciprocally modify neighboring-cell pyroptosis or undergo pyroptosis themselves. Several general interaction motifs have been established in nontumor models. PLAG1-like zinc finger 2 (PLAGL2)–MyD88-dependent hepatocyte pyroptosis increased IL-1β release and activated Janus kinase (JAK)–STAT signaling in BMDMs, whereas pyroptotic trophoblasts and inflammatory macrophages formed a reciprocal amplification circuit in early-onset preeclampsia (140, 141). These studies support paracrine and feedback interactions but cannot be directly generalized to TAMs.

Susceptibility to macrophage pyroptosis is further shaped by metabolic and posttranscriptional control. Interleukin-1 receptor type 2 (IL1R2) restrained enolase 1 (ENO1)-dependent glycolysis and macrophage pyroptosis, glycolysis-derived lactate promoted NLRP3 assembly through alanyl-tRNA synthetase 2 (AARS2)-dependent NLRP3 lactylation, and adenosine deaminase acting on double-stranded RNA 1 (ADAR1) limited NLRP3 activation through a microRNA-21 (miR-21)–TNF alpha-induced protein 3 (TNFAIP3; also known as A20) pathway (142–144). Pyroptosis of donor-derived macrophages also increased CD4^+^ T-cell activation and differentiation in acute graft-versus-host disease (145). Taken together, these findings indicate that macrophage susceptibility to pyroptosis is shaped by metabolic activity, metabolite-dependent protein modification and inhibitory RNA regulatory circuits rather than inflammasome abundance alone.

Tumor models provide more direct evidence of signal decoding and macrophage-intrinsic death. In ovarian cancer, inhibition of splicing factor 3b subunit 1 (SF3B1) induced caspase-3–GSDME-dependent tumor cell pyroptosis; released mitochondrial DNA (mtDNA) was taken up by macrophages and activated cyclic GMP–AMP synthase (cGAS)–stimulator of interferon genes (STING) signaling. Depletion of tumor cell mtDNA weakened this response, supporting a defined pyroptotic cargo–sensor relationship (146). Conversely, a reduction in methionine adenosyltransferase 2A (MAT2A) depleted S-adenosylmethionine (SAM) in BMDMs, induced GSDME-dependent macrophage pyroptosis and enhanced tumor control in the tested murine models (147). Myeloid origin may also influence pathway engagement: in glioma, the expression levels of gasdermin and caspase were greater in infiltrating bone marrow-derived macrophages than in tissue-resident microglia (148). This cell-resolved evidence supports heterogeneity in pyroptosis pathway engagement, although lineage-specific lysis was not established as the cause of immune suppression.

#### 3.3.3 Cellular source and tumor immunity

Macrophage-associated GSDMD–IL-1β signaling has been linked to tumor invasion, although evidence for completed macrophage pyroptosis varies among models. In renal cell carcinoma, macrophage GSDMD increased IL-1β release and promoted tumor cell proliferation, migration and epithelial–mesenchymal transition (EMT), whereas GSDMD inhibition reduced tumor growth and metastasis; terminal macrophage lysis was not directly established (149). In obesity-associated pancreatic cancer, saturated fatty acids increased fatty acid-binding protein 4 (FABP4) in macrophages, induced caspase-1–GSDMD-dependent pyroptosis and promoted EMT, invasion and metastasis through the NLRP3–IL-1β pathway (150). In peripheral T-cell lymphoma, pyroptosis-associated macrophage features were associated with poor prognosis. Mechanistically, IL-18 released from pyroptotic macrophages increased TLR4 expression and downstream NF-κB signaling in lymphoma cells, promoting proliferation and chemoresistance (151). Conversely, MAT2A depletion induced GSDME-dependent macrophage pyroptosis and suppressed tumor growth in murine models (147). Thus, macrophage-derived IL-1β and IL-18 can support invasion or treatment resistance in defined tumor models, whereas MAT2A depletion links GSDME-dependent macrophage pyroptosis to tumor control.

Tumor-cell pyroptosis has been linked to antitumor immune responses. Tumor-cell pyroptosis driven by IKAROS family zinc finger 1 (IKZF1) in colon cancer or guanylate-binding protein 5 (GBP5) in ovarian cancer was accompanied by reduced tumor progression and a shift toward inflammatory macrophage marker expression (152, 153). However, these macrophage-related findings were based mainly on marker expression, coculture, immune-infiltration, and bioinformatic analyses rather than lineage-resolved functional perturbation. In ovarian cancer, macrophage depletion abolished the antitumor effect of pladienolide B-mediated SF3B1 inhibition, supporting a functional requirement for macrophages in this response (146). GSDME-dependent tumor-cell pyroptosis also enhanced TAM phagocytosis and cytotoxic T lymphocyte (CTL) responses, with tumor control requiring CTLs in mouse models (154). In oral squamous cell carcinoma, chemotherapy-induced GSDME-dependent pyroptosis contributed to cisplatin efficacy, and GSDME expression was positively associated with tumor-infiltrating CD8^+^ T cells, granzyme B, and macrophages expressing M1-associated markers. GSDME knockdown increased the cancer stem-cell markers CD44 and aldehyde dehydrogenase 1 (ALDH1) (155).

Pyroptosis in nonmalignant stromal cells can support treatment resistance. In high-grade serous ovarian carcinoma, tumor-derived IL-6 and IL-8 induced NLRP3–caspase-1–GSDMD-dependent pyroptosis in omental adipocytes, and the release of adenosine triphosphate promoted macrophage recruitment and crown-like structure formation, whereas free fatty acids supported tumor cell fatty acid oxidation (FAO) and chemoresistance (156). These structures were associated with shorter progression-free and overall survival, providing complementary but noncausal clinical evidence (157). Thus, stromal-cell pyroptosis can couple ATP-dependent macrophage recruitment to metabolic support of surviving tumor cells.

Taken together, the decisive feature of pyroptosis is the route by which gasdermin-mediated membrane damage is translated into immune signaling. Cytokine release can precede terminal lysis, whereas later membrane disruption exposes intracellular cargo. The cellular source then determines whether macrophages act as recipients of tumor-derived cargo, sources of inflammatory cytokines, or recruited responders to stromal injury.

### 3.4 Autophagy and autophagy-dependent cell death

#### 3.4.1 Autophagy and death-associated signals

Macroautophagy, hereafter referred to as autophagy, is a lysosome-dependent pathway that removes cytoplasmic material and recycles its components. The UNC-51-like autophagy-activating kinase 1 (ULK1) complex and class III phosphatidylinositol 3-kinase complex I (PI3KC3-C1) initiate phagophore formation (158). The autophagy-related protein 12 (ATG12)–ATG5–ATG16L1 complex and lipidation of ATG8 family proteins, including microtubule-associated protein 1 light chain 3 (LC3), subsequently support phagophore expansion and closure (159–161). The fusion of a mature autophagosome with a lysosome results in the formation of an autolysosome, in which proteins, organelles and other cargo are degraded (162, 163).

Autophagy usually supports adaptation to stress and is not, by itself, evidence of cell death (164, 165). Increased LC3-II abundance or autophagosome accumulation may result from increased autophagosome formation or impaired lysosomal turnover. Thus, dynamic measurements of autophagic flux are needed (166). ADCD occurs only when disruption of the autophagy machinery prevents death rather than merely changing cellular stress or another death program (167). LC3-associated phagocytosis (LAP) is also distinct from canonical autophagy. LAP lipidates LC3 on a single-membrane phagosome and does not require the formation of a double-membrane autophagosome (168). Unlike lytic RCD, canonical autophagy is not defined by plasma membrane rupture and does not, by itself, specify a fixed extracellular signal profile (169). In the tumor models discussed below, changes in autophagic flux alter soluble mediators, surface recognition molecules or intracellular cargo available to macrophages. Here, the relevant kinetics include the stage and duration of autophagic flux and the timing of lethal commitment. Death magnitude becomes interpretable only after a causal role for autophagy in lethal execution has been established. Unless stated otherwise, the studies below address autophagy-related regulation rather than ADCD itself.

#### 3.4.2 Autophagy in tumor–macrophage interactions

Autophagy in tumor cells can alter macrophage recruitment and tumor cell recognition. In clear-cell renal cell carcinoma, TRAF2 inhibited tumor cell autophagy and promoted the recruitment of macrophages with an M2-associated phenotype. This pathway also supported angiogenesis and tumor progression (170). Tumor-cell-released autophagosomes (TRAPs) represent a distinct autophagy-associated output. After uptake by macrophages, TRAPs engaged TLR4 and activated downstream MyD88–p38 mitogen-activated protein kinase (p38 MAPK)–STAT3 signaling. These macrophages upregulated programmed death-ligand 1 (PD-L1) and IL-10 and suppressed T-cell proliferation (171). In contrast, autophagy inhibition in PDAC recruited macrophages through C-X-C motif chemokine ligands 1 and 2 (CXCL1/2)–C-X-C motif chemokine receptor 2 (CXCR2) signaling. It also reduced CD47 on tumor cells and increased macrophage-mediated phagocytosis. CD8^+^ T cells supported this phagocytic response rather than acting mainly through direct cytotoxicity (172). These studies suggest that macrophage responses depend on the soluble and surface signals that accompany changes in tumor cell autophagy rather than on autophagy status alone.

Macrophage-intrinsic autophagy and LAP can regulate different stages of signal processing. In hepatocellular carcinoma (HCC), tumor-associated signaling increased mechanistic target of rapamycin (mTOR) activity and inhibitory ULK1 phosphorylation, thereby reducing macrophage autophagy. Reduced autophagic clearance allowed the accumulation of NLRP3 inflammasomes and increased IL-1β maturation. Autophagy inhibition also induced C-C motif chemokine ligand 20 (CCL20)–C-C motif chemokine receptor 6 (CCR6)-dependent self-recruitment by macrophages. Together, these processes promoted epithelial-to-mesenchymal transition and metastasis (173). In acute myeloid leukemia (AML), bone marrow macrophages instead used LAP to process leukemia-derived apoptotic bodies. Mitochondrial DNA within these apoptotic bodies activated STING. This response increased macrophage phagocytosis and restricted leukemia growth (174). These models show that autophagy-related pathways can regulate both the inflammatory machinery and the intracellular processing of engulfed material.

Macrophages can also regulate autophagy in viable tumor cells. In breast cancer, macrophage-derived CXCL1 prevented von Hippel–Lindau protein-dependent ubiquitination and degradation of insulin-like growth factor 1 receptor (IGF1R). The accumulation of IGF1R activated STAT3–HMGB1 signaling, increased protective autophagy and reduced paclitaxel sensitivity (175). In osteosarcoma, macrophage translocase of inner mitochondrial membrane 23 (TIMM23) enhanced mitophagy and promoted an M2-associated program. These macrophages increased TIMM23–PARG pseudogene 1 (PARGP1) fusion transcript expression in tumor cells and reduced chemotherapy sensitivity (176). Both studies concern stress adaptation in viable tumor cells rather than ADCD (177). A recent HCC study further revealed that icaritin-induced downregulation of syntaxin 16 (STX16) impaired autophagosome–lysosome fusion in HCC cells, leading to extracellular-vesicle-mediated transfer of accumulated autophagosomes to macrophages, reduced macrophage viability and sequestosome 1 (SQSTM1; also known as p62)-mediated autophagic degradation of STAT3 (177, 178). Together, these findings define three levels of reciprocal regulation. Autophagy alters the signals available to macrophages. Macrophage autophagy and LAP shape inflammatory signaling and cargo processing. In turn, macrophages can increase protective autophagy and treatment tolerance in viable tumor cells.

#### 3.4.3 Cell-specific effects of autophagy

The outcome of autophagy–macrophage interactions depends on the cell in which autophagy occurs and the macrophage population involved. In ovarian cancer, embryonically derived TIM-4^+^ resident macrophages exhibited high OXPHOS and strong mitophagy. ARG1 decreased intracellular arginine and mechanistic target of rapamycin complex 1 (mTORC1) activity, thereby sustaining mitophagy and limiting mitochondrial ROS. This adaptation supported macrophage survival and peritoneal tumor growth. Human complement receptor of the immunoglobulin superfamily-positive (CRIg^+^) ovarian cancer macrophages exhibited a related transcriptional and metabolic program (179). This receptor-defined resident population provides greater mechanistic resolution than a generic M2 label because its tumor-supporting function is linked to ontogeny, metabolism and mitophagy.

In glioblastoma, imipramine increased autophagy in tumor cells but reprogrammed macrophages through a separate mechanism involving the inhibition of histamine H1 receptor signaling. Concurrent inhibition of the vascular endothelial growth factor (VEGF) pathway remodeled tumor vessels. These parallel effects enabled CD4^+^ and CD8^+^ T-cell infiltration and improved tumor control in mouse models (180). Defined macrophage programs can also shape responses to immunotherapy. In triple-negative breast cancer (TNBC), reduced expression of factor-induced gene 4 (FIG4) impaired the lysosome-associated membrane protein 2A (LAMP2A)-dependent degradation of IL-18. Increased IL-18 secretion promoted the accumulation of immunosuppressive lipid-associated macrophages and resistance to PD-1 blockade. In experimental models, FIG4 overexpression or IL-18 neutralization restored sensitivity to PD-1 blockade (181). These models identify specific molecular control points for macrophage fitness, T-cell access and immunotherapy response. They include ARG1–mTORC1-dependent mitophagy, histamine H1 receptor signaling, vascular access and FIG4–LAMP2A-dependent IL-18 degradation.

Tumor-cell and macrophage autophagy have different consequences in the models reviewed here. Inhibiting tumor cell autophagy enhanced macrophage phagocytosis in pancreatic cancer, whereas loss of macrophage autophagy amplified inflammasome signaling and metastasis in HCC (172, 173). The findings in ovarian and breast cancer models further revealed that macrophage mitophagy or macrophage-induced tumor cell autophagy can support tumor growth and treatment resistance (175, 179). These mechanisms predict different consequences of nonselective autophagy inhibition. It may weaken tumor cell stress adaptation while disrupting macrophage cargo processing, inflammatory restraint or metabolic fitness (182–184). Therapeutic studies should therefore define the target cell, the stage and duration of autophagy perturbation and the macrophage function being measured. Mechanistic causality has been established mainly in cell-based and mouse tumor models, although several studies include supportive analyses of human samples. Most studies address autophagy-related regulation of survival, inflammation or phagocytosis rather than completed ADCD. Direct evidence that completed ADCD generates a distinct macrophage response in tumors remains limited. Accordingly, the kinetics and magnitude of confirmed ADCD have not been quantitatively linked to macrophage functional states in tumors. Future studies should pair time-resolved measurements of autophagic flux and lethal commitment with lethal fraction, signal availability and macrophage functional readouts.

### 3.5 Ferroptosis

#### 3.5.1 Lipid peroxidation and signal exposure

Ferroptosis is an iron-dependent RCD program caused by uncontrolled phospholipid peroxidation. Acyl-CoA synthetase long chain family member 4 (ACSL4) and lysophosphatidylcholine acyltransferase 3 (LPCAT3) are enriched in cellular membranes with oxidation-prone polyunsaturated fatty acid-containing phospholipids (PUFAs) (185), whereas the cystine–glutathione–glutathione peroxidase 4 (GPX4) axis removes phospholipid hydroperoxides (186). Ferroptosis suppressor protein 1 (FSP1)–coenzyme Q10 (CoQ10) and GTP cyclohydrolase 1 (GCH1)–tetrahydrobiopterin (BH4) provide parallel antioxidant protection (187, 188). Iron uptake and nuclear receptor coactivator 4 (NCOA4)-dependent ferritinophagy can lower the ferroptotic threshold by increasing the labile iron pool (189, 190), but these processes regulate susceptibility rather than constitute universal terminal execution steps (191).

Signal exposure changes as ferroptotic injury progresses. Before terminal membrane failure, oxidized phosphatidylethanolamines such as 1-stearoyl-2-(15-hydroperoxyeicosatetraenoyl)-sn-glycero-3-phosphatidylethanolamine (SAPE-OOH) can become accessible on the cell surface and support phagocytic recognition (192). Progressive membrane damage subsequently increases extracellular access to nucleotides, proteins and other intracellular material (193–195). In a murine vaccination model, early ferroptotic cancer cells induced protective antitumor immunity, whereas later-stage ferroptotic cells did not produce an equivalent response (69). Thus, the stage of ferroptotic execution determines which signals are accessible and when they become available to immune cells. Completed ferroptosis, DAMP release and immunogenic cell death remain distinct experimental endpoints (196).

#### 3.5.2 Macrophage recognition and control of ferroptosis

Macrophages interpret ferroptotic cells through distinct recognition and intracellular-processing routes. Surface SAPE-OOH bound to TLR2 and promoted the uptake of ferroptotic cells (192). In contrast, autophagy-dependent ferroptosis in PDAC promoted the exosomal release of oncogenic KRAS^G12D, which was internalized through the receptor for advanced glycation end products (RAGE) and activated STAT3-dependent FAO in macrophages (197). These findings distinguish corpse recognition from cargo interpretation: surface oxidized lipids can facilitate the capture of ferroptotic material, whereas internalized molecular cargo can reprogram macrophage metabolism after uptake.

The state of the recipient macrophage affects both its susceptibility to ferroptotic stress and its ability to clear ferroptotic cells. Under defined lipopolysaccharide and interferon-γ stimulation, inducible nitric oxide synthase (iNOS)-derived nitric oxide modified proferroptotic phospholipids and protected macrophages from ferroptotic death (198). More recent work has shown that lipopolysaccharide- and lipopolysaccharide–interferon-γ-stimulated macrophages use distinct GCH1–BH4 and iNOS programs, respectively, to resist ferroptosis and that these protective states are reversible after the activation of these stimuli is withdrawn (199). However, in TAMs exposed to phospholipid peroxidation, SAPE-OOH disrupted the interaction between TLR2 and its chaperone canopy FGF signaling regulator 3 (CNPY3), causing the retention of TLR2 in the endoplasmic reticulum (ER) and subsequent membrane-associated ring-CH-type finger 6 (MARCH6)-dependent proteasomal degradation. The resulting loss of surface TLR2 reduced the clearance of ferroptotic tumor cells (200). Single-cell and spatial analyses have additionally identified iron-rich TAMs comprising transcriptionally and spatially distinct perivascular and stromal states, demonstrating heterogeneity in iron handling within the tumor macrophage compartment (201). Together, these studies support state-dependent variation in macrophage redox defense, iron handling and phagocytic competence rather than a fixed M1/M2 identity. However, neither the iron-rich tumor-associated macrophage study nor the available SAPE-OOH–TLR2 studies mapped ferroptotic cell recognition and clearance across contemporary marker-defined tumor-associated macrophage subsets.

Macrophages also modify the ferroptotic threshold of neighboring tumor cells. Direct coculture has shown that macrophages reduced lipid peroxidation and rescued several cell types during the early, contact-dependent phase of ferroptotic stress (202). Conversely, tumor necrosis factor-α (TNF-α) released from experimentally M1-polarized macrophages activated TNFR1–p38 MAPK signaling, increased ACSL4 expression and promoted ferroptosis in pancreatic cancer cells (203). The timing of macrophage input is important in these models. Contact-dependent protection acts during early reversible lipid damage, whereas TNF-α signaling increases ACSL4 expression and lowers the threshold for ferroptotic execution.

#### 3.5.3 Macrophage metabolism and treatment response

Ferroptosis–macrophage coupling promotes tumor progression most clearly when a shared metabolic circuit simultaneously protects tumor cells and reprograms macrophages. In HCC, transmembrane protein 147 (TMEM147) increased 27-hydroxycholesterol (27HC) production through STAT2 and 7-dehydrocholesterol reductase (DHCR7). The metabolite increased tumor cell GPX4 while activating peroxisome proliferator-activated receptor gamma (PPARγ)-dependent FAO in macrophages, thereby promoting tumor invasion (204). In colorectal cancer, inhibin subunit beta A (INHBA) stabilized mitochondrial solute carrier family 25 member 10 (SLC25A10), reinforced the mitochondrial glutathione–GPX4 defense and activated succinate receptor 1 (SUCNR1) signaling in macrophages (205). Both studies established coordinated tumor cell redox protection and macrophage metabolic reprogramming rather than a simple linear sequence from ferroptosis inhibition to M2 polarization.

Macrophage redox defense and antigen processing represent separate immune control points during ferroptotic stress. In HCC, apolipoprotein C1 (APOC1) inhibition increased iron and ROS levels in TAMs, weakened the nuclear factor erythroid 2–related factor 2 (NRF2)–solute carrier family 7 member 11 (SLC7A11)–GPX4 defense and altered the macrophage-state composition. These changes were accompanied by greater CD8^+^ T-cell and NK-cell infiltration and improved anti-PD-1 efficacy in mice (206). Consistently, macrophage-specific SLC7A11 deletion increased the ferroptotic susceptibility of TAMs, reduced their M2-like protumoral polarization and accumulation, and enhanced the efficacy of anti-PD-L1 therapy in HCC models (207). A tumor-selective nanoplatform study further revealed that all-trans retinoic acid (ATRA) released from ferroptotic tumor cells activated retinoic acid receptor alpha (RARα)–CD38–transcription factor EB (TFEB) signaling and increased major histocompatibility complex class II (MHC-II)-dependent antigen presentation by tumor-infiltrating macrophages (208). Together, the first two studies support the ferroptotic vulnerability of protumoral TAM states and its therapeutic relevance, whereas the nanoplatform study links a defined metabolite released by ferroptotic tumor cells to macrophage antigen processing.

Therapeutic resistance can arise through local rescue, impaired corpse clearance and vesicle-mediated redistribution of iron and antioxidant capacity. Contact-dependent protection may preserve tumor cells before lipid damage becomes irreversible (202), whereas phospholipid peroxidation in macrophages can impair TLR2-dependent removal after ferroptosis has occurred (200). Tumor-associated macrophage-derived extracellular vesicles can further transfer iron-regulatory and antioxidant cargo to residual tumor cells, extending ferroptosis resistance beyond direct cell contact (209, 210).

Ferroptosis is distinguished by a lipid-redox interface between dying tumor cells and macrophages. Before lipid peroxidation becomes irreversible, macrophages can rescue stressed tumor cells. As oxidation progresses, surface SAPE-OOH promotes TLR2-dependent uptake, whereas phospholipid peroxidation in macrophages reduces surface TLR2 and impairs clearance. The relevant magnitude may therefore reflect the local burden of oxidized phospholipids relative to macrophage antioxidant and phagocytic capacity, rather than simply the fraction of tumor cells that die. This relationship has not been quantified in tumors. Defining when ferroptotic lipid signals shift from supporting recognition to impairing macrophage function may help optimize the timing and intensity of ferroptosis-based therapy.

### 3.6 Cuproptosis

#### 3.6.1 Copper-dependent death and signal release

Cuproptosis is a copper-dependent RCD program that requires mitochondrial respiration and protein lipoylation. Excess copper binds lipoylated tricarboxylic acid (TCA) cycle proteins, particularly dihydrolipoamide S-acetyltransferase (DLAT), causing lipoylated protein aggregation, loss of iron–sulfur cluster proteins and proteotoxic stress (211). Ferredoxin 1 (FDX1) and the mitochondrial lipoylation machinery are key determinants of whether copper accumulation is converted into this lethal state (212). In breast cancer, mitochondrion-targeted copper dithiocarbamate induced cuproptosis together with DAMP release (213). In a tumor-targeted nanoregulator system, combined delivery of a copper ionophore and ATG5-directed autophagy blockade amplified tumor-cell cuproptosis, accompanied by calreticulin (CRT) exposure and extracellular HMGB1 release (214). These models link experimentally induced cuproptosis to CRT exposure and HMGB1 release, but the timing of these signals relative to lethal commitment remains unresolved. Copper accumulation, mitochondrial injury and cuproptosis-related transcriptional scores therefore remain insufficient evidence of completed cuproptosis (215, 216).

#### 3.6.2 Macrophage responses and copper metabolism

Macrophages respond to the specific signals made accessible during tumor cell cuproptosis. In breast cancer, mitochondrion-targeted copper dithiocarbamate induced cuproptosis and DAMP release, which increased macrophage migration and the expression of M1-associated markers (213). In NSCLC, copper-induced CRT exposure and ATP and HMGB1 release were accompanied by a proinflammatory macrophage shift. Pharmacological suppression of cuproptosis reduced these immunogenic outputs (217). In cervical cancer, depletion of NIMA-related kinase 3 (NEK3) increased sensitivity to elesclomol–copper and promoted macrophage recruitment and an M1-associated program through MAPK signaling (218). These models identify CRT, ATP, HMGB1 and MAPK signaling as molecular links between tumor cell cuproptosis and macrophage inflammatory reprogramming.

Copper metabolism within macrophages can also alter their functional state and tumor cell susceptibility to death. Outside tumor models, CD44-mediated copper uptake generated a reactive mitochondrial copper(II) pool that maintained nicotinamide adenine dinucleotide availability and supported inflammatory metabolic and epigenetic programs without causing macrophage death (219). In TNBC, solute carrier family 31 member 1 (SLC31A1)-high macrophages contained more copper and hydrogen peroxide and expressed more M2-associated markers. SLC31A1 knockdown reduced these features, and conditioned medium from the modified macrophages inhibited tumor cell migration and invasion while increasing apoptosis (220). Glycine cleavage system protein H-positive (GCSH^+^) macrophages were enriched at the tumor core–stroma interface and were associated with altered amino acid and lipid metabolism, immunoregulatory communication, poor outcomes and chemotherapy resistance (221). Conversely, phosphatidylinositol-4,5-bisphosphate 3-kinase catalytic subunit gamma *(Pik3cg)* editing in tumor-associated macrophages drove M2-to-M1 repolarization and increased tumor cell copper accumulation through a CD44–hyaluronic acid (HA)-associated copper uptake pathway, thereby enhancing cuproptosis (222). Thus, macrophage copper uptake and metabolic state can regulate both macrophage polarization and the cuproptotic threshold of neighboring tumor cells.

#### 3.6.3 Tumor immunity and treatment resistance

The tumor outcome depends on whether copper stress progresses to completed tumor cell cuproptosis and on the state of macrophages that receive or modify the resulting signals. Experimental interventions that increased cuproptosis-associated tumor cell death in cervical and NSCLC reduced tumor growth and were accompanied by macrophage recruitment or proinflammatory macrophage features (217, 218). Functional perturbation of SLC31A1-high macrophages altered tumor cell migration, invasion and apoptosis (220), whereas GCSH^+^ macrophages were identified mainly through integrated single-cell, spatial and clinical association analyses and were associated with immunoregulatory signaling, poor outcomes and chemotherapy resistance (221). These findings describe different biological processes rather than opposite consequences of the same event. The first group tested experimentally induced tumor cell death, whereas the second characterized viable macrophage populations with altered copper metabolism. Therefore, cuproptosis-related gene expression in macrophages should not be equated with macrophage death or antitumor activity.

Cuproptosis can also influence the response to immunotherapy, although the contribution of macrophages should be separated from parallel effects on dendritic cells and lymphocytes. In NSCLC, copper-induced cuproptosis promoted dendritic cell maturation, reduced myeloid-derived suppressor-cell accumulation, increased T-cell infiltration and improved programmed death-ligand 1 blockade, together with the macrophage changes described above (217). A separate study revealed that DAMPs released by cuproptotic tumor cells activated dendritic cells, whereas interferon-γ from CD8^+^ T cells increased tumor cell FDX1 transcription through signal transducer and activator of transcription 1–interferon regulatory factor 1 signaling. This reciprocal pathway increased tumor cell susceptibility to cuproptosis and improved the response to programmed death-ligand 1 blockade in preclinical models (223). These studies provide preclinical evidence of immune feedback around cuproptosis, but only the former directly measured macrophage responses.

**Figure 2:**
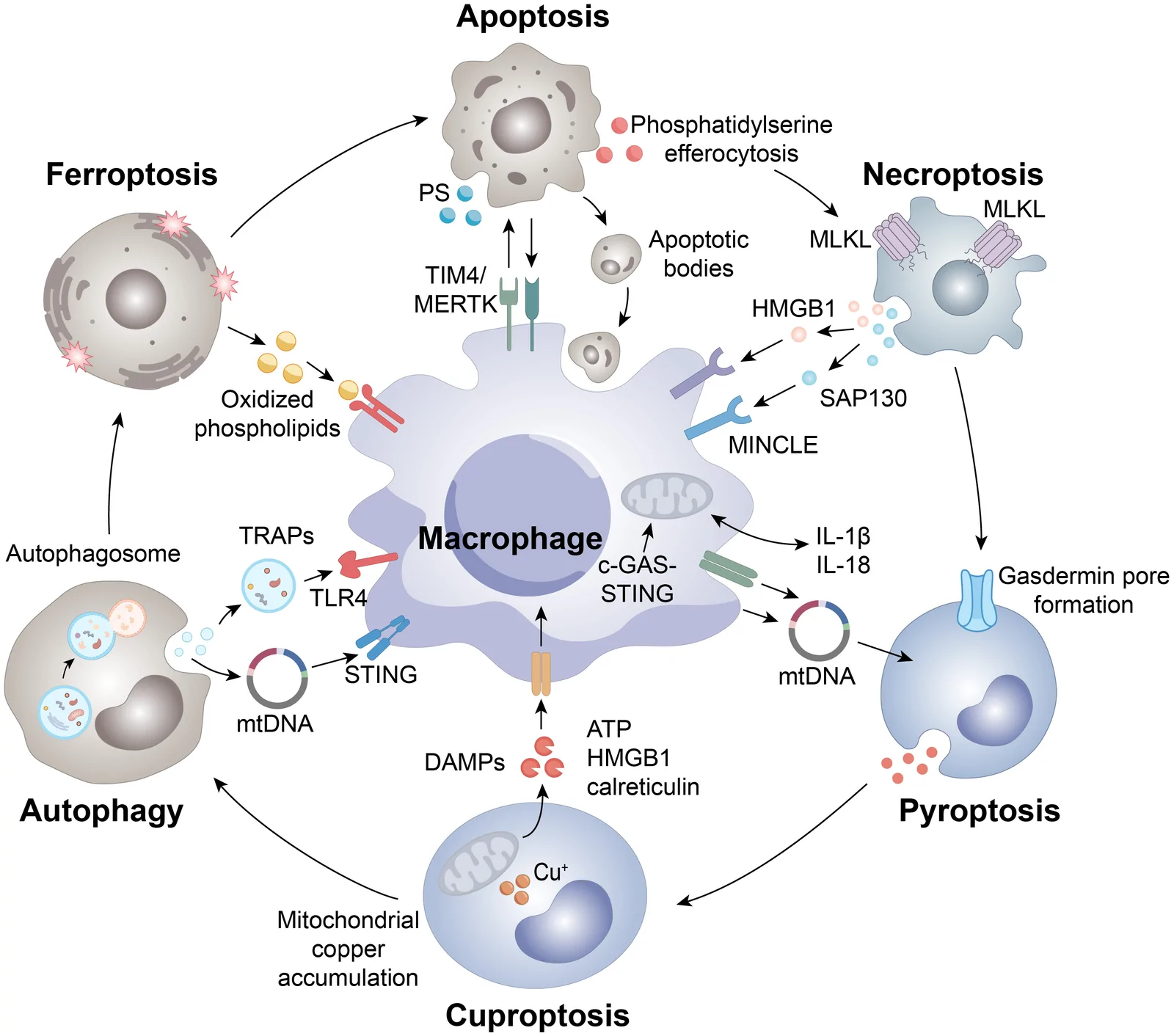
Molecular decoding of signals released from distinct regulated cell death modalities by macrophages. Different RCD programmes generate character**i**stic molecular outputs that are sensed and processed by macrophages through partially overlapping but mechanistically distinct pathways. Apoptotic cells expose phosphatidylserine and are removed through efferocytic receptors such as TIM4 and MERTK, whereas necroptotic cells release intracellular components including HMGB1 and SAP130 following MLKL-dependent membrane disruption. Pyroptotic cells generate gasdermin-mediated pores and release inflammatory cytokines, including IL-1β and IL-18, together with mitochondrial DNA that can activate innate immune sensing pathways. Ferroptotic cells expose oxidized phospholipids and release lipid-associated signals that influence macrophage recognition and metabolic reprogramming. Autophagy-related pathways modify macrophage responses through extracellular vesicles, autophagosome-derived cargo and altered intracellular processing. Cuproptotic tumor cells release immunogenic stress signals, including ATP, HMGB1 and calreticulin, although the specificity of these signals requires further investigation. Collectively, macrophages function as signal-decoding hubs that integrate diverse death-associated inputs rather than responding to individual RCD modalities as isolated events.

Therapeutic resistance can arise before macrophage decoding occurs because tumor cells may remain below the cuproptotic threshold. Under hypoxia, hypoxia-inducible factor 1 alpha (HIF-1α) activated pyruvate dehydrogenase kinases 1 and 3, reduced DLAT abundance and increased metallothionein-mediated sequestration of mitochondrial copper, thereby limiting cuproptosis (224). In lung adenocarcinoma, nicotine maintained a cuproptosis-resistant lipoic acid synthetase–DLAT metabolic state and promoted the M2-associated macrophage phenotype, as measured in that study (225). These findings suggest that tumor cell metabolic adaptation and tumor-supporting macrophage programs can coexist within the same microenvironment, although they do not establish a universal causal pathway between the two. Overall, the cuproptosis–macrophage axis is shaped by both the tumor cell execution threshold and the copper metabolic state of the responding macrophage. Mechanistic evidence derives mainly from cell-based and mouse tumor models. Human single-cell and spatial evidence remains predominantly associative, although some studies include complementary *in vitro* perturbation experiments (**Figure 2**; **Table 1**).

**Table 1:** Representative context-dependent interactions between regulated cell death signals and macrophages in cancer.

RCD modality	Cell type undergoing RCD/direction	Macrophage subset	Cancer type	Key mediators/signals	Experimental model	Evidence level	Functional outcome/therapeutic relevance	Ref.
Apoptosis	PS-bearing tumor material → macrophage	TIM-4^+^ large resident peritoneal macrophages	Ovarian cancer (OC)	PS, TIM-4, F-actin, v-ATPase	Cell assays, syngeneic OC tumor	Causal preclinical	Preserves tumor antigens for cross-presentation, enhances CD8^+^ T-cell responses, and restrains early OC growth; Not tested	(92)
	CD169^^+^^ macrophage → tumor cell	CD169^+^ macrophages	Colorectal cancer (CRC)	CD169, CD43, FasL, Fas	Coculture, CRC mouse models, patient cohort	Causal preclinical + human associative	Promotes tumor-cell apoptosis and suppresses CRC growth; associated with longer survival; Not tested	(100)
	Therapy-killed tumor debris → lymph-node macrophage	Medullary sinus macrophages	Melanoma (MEL), other solid-tumor models	IL-33, ST2	TDLN mouse models, human	Causal preclinical + human supportive	Activates ST2^+^ Tregs, suppresses CD8^+^ T-cell activity, and promotes treatment resistance; ST2 blockade improves preclinical treatment response	(106)
Necroptosis	Tumor-cell RIPK1/RIPK3 signaling → macrophage/myeloid cells	Tumor-infiltrating macrophages/myeloid cells	Pancreatic ductal adenocarcinoma (PDAC)	RIPK1, RIPK3, CXCL1, SAP130, MINCLE	Genetic and orthotopic PDAC models	Mechanistic preclinical	Promotes macrophage-mediated adaptive immune suppression and PDAC progression; RIPK/CXCL1/MINCLE pathway blockade shows preclinical activity	(117)
	Macrophage → viable tumor cell	M2-like TAMs	Colorectal cancer (CRC)	METTL3, m6A, TRAF5	Coculture, oxaliplatin CRC	Mechanistic preclinical + human associative	Suppresses CRC-cell necroptosis and confers oxaliplatin resistance; TAM/METTL3 targeting shows preclinical relevance	(121)
	Tumor endothelial cell → macrophage	CXCL10^+^ macrophages	Ovarian cancer (OC); breast cancer (BC)	PARP1, MLKL, CXCL12, CXCR4, CXCL10, CXCR3	Chemotherapy-treated OC/BC mouse models, patient samples	Causal preclinical + human supportive	Suppresses the CXCL10^^+^^ macrophage–CXCR3^+^ CD8^^+^^ T-cell axis and promotes chemotherapy resistance; CXCL12/CXCR4 blockade partially restores antitumor immunity preclinically	(125)
Pyroptosis	Pyroptotic tumor cell → macrophage	M1-like macrophages	Ovarian cancer (OC)	SF3B1, BCL2L2, GSDME, mtDNA, cGAS, STING	Cell assays; syngeneic OC tumor model, datasets	Causal preclinical + human associative	Activates macrophage cGAS–STING signaling and strengthens antitumor immunity; SF3B1 inhibition enhances anti-PD-L1 control preclinically	(146)
	Macrophage-intrinsic pyroptosis	BMDMs and tumor-infiltrating macrophages	Melanoma (MEL); other syngeneic tumors	MAT2A, GSDME	Myeloid Mat2a deletion, syngeneic tumor models	Causal preclinical	Promotes GSDME-dependent macrophage pyroptosis and antitumor immunity, suppressing tumor growth; MAT2A inhibition shows preclinical potential	(147)
	omental adipocyte → macrophage/tumor cell	Recruited omental/CLS-associated macrophages	Ovarian cancer (OC)	IL-6, IL-8, GSDMD, ATP, FFA	Coculture, adipocyte organoids, OC mouse/omentum analyses	Causal preclinical + human associative	Promotes macrophage/CLS recruitment, tumor metabolism, invasion, and chemoresistance; Disulfiram shows preclinical activity	(156)
Autophagy-related regulation	Tumor-cell-released autophagosome → macrophage	M2-like macrophages	Melanoma (MEL)	TLR4, MyD88, p38, STAT3, PD-L1, IL-10	Macrophage assays, B16F10 MEL model, patient fluid	Causal preclinical + ex vivo human	Induces immunosuppressive macrophages, suppresses T-cell proliferation, and accelerates MEL growth; TRAPs/PD-L1 blockade shows preclinical relevance	(171)
	Tumor environment → macrophage-intrinsic autophagy	HCC-associated macrophages	Hepatocellular carcinoma (HCC)	mTOR, ULK1, NLRP3, IL-1β, CCL20, CCR6	Cell assays, HCC metastasis model, patient tissue	Causal preclinical + human associative	Promotes macrophage self-recruitment, HCC EMT, invasion, and lung metastasis; IL-1β blockade shows preclinical relevance	(173)
	Macrophage → viable tumor cell	M2-like TAMs	Breast cancer (BC)	CXCL1, VHL, IGF1R, STAT3, HMGB1	Coculture, BC xenograft, tissue array	Causal preclinical + human associative	Induces protective tumor-cell autophagy and reduces paclitaxel sensitivity in BC; CXCL1/autophagy targeting shows preclinical relevance	(175)
Ferroptosis	Ferroptotic tumor cell → macrophage	M2-like macrophages	Pancreatic ductal adenocarcinoma (PDAC)	KRAS^^^G12D, AGER/RAGE, STAT3	Exosome assays, PDAC mouse model, survival data	Causal preclinical + human associative	Induces an M2-like macrophage program and accelerates PDAC growth; Blocking KRAS^G12D release or uptake shows preclinical potential	(197)
	Macrophage phospholipid peroxidation → impaired ferroptotic-cell clearance	Tumor-microenvironment macrophages	Breast cancer (BC)	SAPE-OOH, TLR2, CNPY3, MARCH6	Myeloid Gpx4 loss, EO771 BC model, lipidomics	Causal preclinical	Impairs ferroptotic-cell clearance and confers resistance to ferroptosis therapy; The TLR2–MARCH6 axis is a potential preclinical target	(200)
	Ferroptotic tumor cell → macrophage	MHC-II-high macrophages	Colorectal cancer (CRC); hepatocellular carcinoma (HCC)	ATRA, RARα, CD38, TFEB, MHC-II	Multi-omics, CRC/HCC mouse models, ICB cohorts	Causal preclinical + human associative	Enhances macrophage MHC-II-dependent antigen presentation; The ferroptosis platform enhances anti-PD-1 control preclinically	(208)
Cuproptosis	Cuproptotic tumor cell → macrophage	M1-like macrophages	Non-small-cell lung cancer (NSCLC)	Cu²^+^, CRT, ATP, HMGB1	NSCLC cell and mouse models	Mechanistic preclinical	Promotes pro-inflammatory macrophage features following tumor-cell cuproptosis; Copper treatment enhances anti-PD-L1 control preclinically	(217)
	Cuproptotic tumor stress → macrophage	M1-like macrophages	Cervical cancer (CC)	NEK3, ROS, MAPK	Multi-omics, coculture, CC xenograft	Mechanistic preclinical	Promotes macrophage recruitment and M1-like features, with reduced CC growth and invasion; Combined NEK3 targeting and elesclomol–Cu shows preclinical efficacy	(218)
	Engineered TAM → tumor cell	M2-like TAMs	Triple-negative breast cancer (TNBC)	Pik3cg/PI3Kγ, CD44, HA, Cu²^+^	CRISPR-programmed biohybrid, 4T1 TNBC model	Mechanistic preclinical	Repolarizes engineered TAMs and enhances tumor-cell cuproptosis/ICD, suppressing TNBC growth and metastasis; Preclinical engineered strategy; endogenous relevance remains unestablished	(222)

AGER, advanced glycosylation end-product specific receptor; ATRA, all-trans retinoic acid; ATP, adenosine triphosphate; BC, breast cancer; BCL2L2, BCL2 like 2; BMDMs, bone marrow-derived macrophages; CC, cervical cancer; CCL20, C-C motif chemokine ligand 20; CCR6, C-C motif chemokine receptor 6; CD8, cluster of differentiation 8; CD38, cluster of differentiation 38; CD43, cluster of differentiation 43; CD44, cluster of differentiation 44; CD169, sialoadhesin (SIGLEC1; sialic acid binding Ig like lectin 1); cGAS, cyclic GMP–AMP synthase; CLS, crown-like structures; CNPY3, canopy FGF signaling regulator 3; CRC, colorectal cancer; CRISPR, clustered regularly interspaced short palindromic repeats; CRT, calreticulin; Cu, copper; Cu²^+^, copper(II) ion; CXCL1, C-X-C motif chemokine ligand 1; CXCL10, C-X-C motif chemokine ligand 10; CXCL12, C-X-C motif chemokine ligand 12; CXCR3, C-X-C motif chemokine receptor 3; CXCR4, C-X-C motif chemokine receptor 4; EMT, epithelial–mesenchymal transition; F-actin, filamentous actin; Fas, Fas cell surface death receptor; FasL, Fas ligand; FFA, free fatty acids; Gpx4, glutathione peroxidase 4 (mouse gene symbol); GSDMD, gasdermin D; GSDME, gasdermin E; HA, hyaluronic acid; HCC, hepatocellular carcinoma; HMGB1, high-mobility group box 1; ICB, immune checkpoint blockade; ICD, immunogenic cell death; IGF1R, insulin-like growth factor 1 receptor; IL-1β, interleukin-1β; IL-6, interleukin-6; IL-8, interleukin-8; IL-10, interleukin-10; IL-33, interleukin-33; KRAS^G12D, KRAS proto-oncogene, GTPase G12D (Gly12Asp) mutant; M1-like, macrophages with M1-associated features; M2-like, macrophages with M2-associated features; MAPK, mitogen-activated protein kinase; MARCH6, membrane-associated ring-CH-type finger 6; MAT2A, methionine adenosyltransferase 2A; Mat2a, methionine adenosyltransferase 2A (mouse gene symbol); MEL, melanoma; METTL3, methyltransferase-like 3; MHC-II, major histocompatibility complex class II; MINCLE, macrophage-inducible C-type lectin; MLKL, mixed lineage kinase domain-like pseudokinase; m6A, N6-methyladenosine; mtDNA, mitochondrial DNA; mTOR, mechanistic target of rapamycin; MyD88, myeloid differentiation primary response 88; NEK3, NIMA-related kinase 3; NLRP3, NLR family pyrin domain containing 3; NSCLC, non-small cell lung cancer; OC, ovarian cancer; p38 MAPK, p38 mitogen-activated protein kinase; PARP1, poly(ADP-ribose) polymerase 1; PD-1, programmed cell death protein 1; PD-L1, programmed death-ligand 1; PDAC, pancreatic ductal adenocarcinoma; PI3Kγ, phosphatidylinositol-4,5-bisphosphate 3-kinase catalytic subunit gamma; Pik3cg, phosphatidylinositol-4,5-bisphosphate 3-kinase catalytic subunit gamma (mouse gene symbol); PS, phosphatidylserine; RAGE, receptor for advanced glycation end products; RARα, retinoic acid receptor alpha; RCD, regulated cell death; RIPK, receptor-interacting protein kinase; RIPK1, receptor-interacting protein kinase 1; RIPK3, receptor-interacting protein kinase 3; ROS, reactive oxygen species; SAP130, spliceosome-associated protein 130; SAPE-OOH, 1-stearoyl-2-(15-hydroperoxyeicosatetraenoyl)-sn-glycero-3-phosphatidylethanolamine; SF3B1, splicing factor 3b subunit 1; ST2, interleukin 1 receptor-like 1 (IL1RL1; also known as ST2); STAT3, signal transducer and activator of transcription 3; STING, stimulator of interferon genes; TAM/TAMs, tumor-associated macrophage(s); TDLN, tumor-draining lymph node; TFEB, transcription factor EB; TIM-4, T-cell immunoglobulin and mucin domain-containing protein 4; TLR2, Toll-like receptor 2; TLR4, Toll-like receptor 4; TNBC, triple-negative breast cancer; TRAF5, TNF receptor-associated factor 5; TRAPs, tumor-cell-released autophagosomes; Tregs, regulatory T cells; ULK1, UNC-51-like autophagy-activating kinase 1; v-ATPase, vacuolar-type H^+^-ATPase; VHL, von Hippel–Lindau tumor suppressor.

## 4 Regulated cell death: comparison and crosstalk

### 4.1 Comparison of cell death modalities

The six RCD modalities are distinguished by the molecular machinery that executes cellular demise. Apoptosis proceeds through caspase-dependent proteolysis, whereas necroptosis and pyroptosis rely on activated MLKL and gasdermin pores, respectively (226). Ferroptosis instead results from iron-dependent phospholipid peroxidation (227). Cuproptosis occurs when copper binds lipoylated TCA-cycle proteins, causing protein aggregation, loss of iron–sulfur proteins, and proteotoxic stress (211). ADCD requires a causal contribution from the autophagic machinery; increased autophagic flux alone is insufficient for this classification (228). These mechanisms generate distinct membrane trajectories and signal profiles. Apoptotic cells generally retain membrane integrity during early execution. This permits the exposure of surface clearance cues and the selective release of metabolites without wholesale leakage of cytosolic contents (87, 229). Necroptosis and pyroptosis result in earlier membrane permeabilization and progress toward terminal rupture, which broadens the extracellular availability of intracellular material (230). GSDMD pores may also permit IL-1β release before complete lysis (231). Ferroptosis culminates in membrane failure after lipid peroxidation has established a lethal state, with ninjurin-1 (NINJ1) contributing to subsequent rupture and DAMP release (232). Its signal profile is further distinguished by oxidized phospholipids, including surface-associated SAPE-OOH (192). Comparable extracellular signatures specific to ADCD or cuproptosis remain less clearly characterized (9, 233).

These differences influence inflammatory potential but do not predetermine antitumor immunity. Selectively induced necroptosis generated type I interferon-dependent tumor protection in a prophylactic model (234). Engineered amplification of tumor cell pyroptosis also strengthened antitumor responses in preclinical settings (235). Conversely, interleukin-1 alpha (IL-1α) released during necrotic-like tumor cell death can establish a suppressive myeloid environment and restrict T-cell activity (236). Ferroptotic tumor cells can likewise be poorly engulfed and impair dendritic cell maturation and antigen cross-presentation (237). Thus, inflammatory intensity should not be regarded as a surrogate for productive antitumor immunity. Although the six RCD modalities are defined by distinct execution mechanisms, their immunological boundaries are less rigid (238). Overlapping stress responses, protease networks, organelle stress, and metabolic perturbations can redirect death trajectories or coactivate multiple execution programs within the same tumor ecosystem (239–241). Such interactions alter the composition and timing of signals emitted by dying tumor cells, thereby shaping how macrophages detect, process, and feedback on subsequent cell death responses (242).

Across RCD modalities, the immune relevance of timing and magnitude depends on when and how death-associated material becomes available to macrophages. In apoptosis, macrophage efferocytosis can prevent necrosis and inflammation under conditions of increased apoptotic-cell burden (243). In necroptosis, plasma membrane rupture occurs downstream of MLKL activation and is mediated by SIGLEC12 in human cells (244). In pyroptosis, GSDMD pores mediate cytokine release, whereas NINJ1 mediates a distinct plasma membrane rupture step (139, 245). In ferroptosis, lipid peroxidation follows a temporal progression, with oxidized lipids moving from intracellular membranes toward the plasma membrane during ferroptosis (246). For ADCD and cuproptosis, the studies reviewed here do not yet define comparable quantitative relationships between death kinetics or magnitude and macrophage responses. Thus, the amount of tumor cell death is best interpreted in relation to death kinetics, membrane integrity, signal persistence, and macrophage processing capacity.

### 4.2 Crosstalk in the macrophage–tumor ecosystem

RCD programs are mechanistically distinct but interconnected through checkpoints that redirect their terminal outputs. Caspase-8 promotes death-receptor-mediated apoptosis while restraining RIPK1–RIPK3 activation and RIPK3–MLKL-dependent necroptosis. In a caspase-8 autocleavage-defective knock-in model, impaired RIPK1 cleavage redirected TNF-α-induced apoptosis toward RIPK3–MLKL-dependent necroptosis (247). GSDME provides a second switch: in GSDME-high tumor cells, ionizing radiation activated the caspase-9–caspase-3–GSDME axis, causing membrane permeabilization and pyroptotic morphology (248). Clearance failure results in a non-cell-autonomous transition. In preclinical tumor models, inhibition of phosphatidylserine-dependent efferocytosis prevented the removal of apoptotic cancer cells and promoted secondary necrosis with increased release of intracellular material (249). NINJ1 adds a final regulatory layer because its blockade reduced terminal plasma membrane rupture and the levels of circulating HMGB1 and IL-18 (250). Thus, pathway selection, corpse clearance and membrane rupture jointly determine whether dying-cell contents remain compartmentalized or become extracellularly available.

Autophagy connects metabolic RCD programs mainly by regulating substrate availability and stress tolerance. The clearest example is NCOA4-dependent ferritinophagy. Upregulation of prostate apoptosis response-4 (Par-4; encoded by PAWR) promoted NCOA4-mediated ferritin degradation, increased labile iron and lipid peroxidation, and sensitized tumor cells to ferroptosis; moreover, Par-4 depletion attenuated ferroptosis-mediated tumor suppression in xenografts (251). Ferritinophagy can also support tumor survival. In PDAC, NCOA4-dependent ferritin turnover sustained iron availability for iron–sulfur-cluster proteins, preserved mitochondrial homeostasis and promoted tumor progression (252). Autophagic degradation also restrains inflammatory signaling: palmitoylation of NLR family pyrin domain-containing protein 3 (NLRP3) promoted its chaperone-mediated autophagic degradation and limited sustained inflammasome activation (253). Cuproptosis remains mechanistically distinct. Copper binding to lipoylated TCA-cycle proteins caused protein aggregation, loss of iron–sulfur-cluster proteins and proteotoxic stress (254, 255). Current evidence has established a direct ferritinophagy–ferroptosis axis. A bona fide endogenous switch between ferroptosis and cuproptosis has not yet been demonstrated; however, pharmacological crosstalk has been reported, as ferroptosis inducers such as sorafenib and erastin can potentiate copper ionophore-induced cuproptosis by reducing glutathione availability and stabilizing FDX1-dependent protein lipoylation. These findings indicate pathway cooperation rather than confirmed conversion from one death modality into the other (256).

These interactions are important because they alter the signals processed by macrophages. Conversion of apoptosis to GSDME-dependent pyroptosis or secondary necrosis increased the extracellular availability of intracellular material within the tumor microenvironment (248). Ferroptosis generates a distinct reciprocal interaction. In TAMs, phospholipid peroxidation disrupted the interaction between TLR2 and CNPY3, retained TLR2 in the ER and promoted MARCH6-dependent degradation. The loss of TLR2 impaired the clearance of ferroptotic tumor cells and promoted resistance to ferroptosis-inducing therapy (200). Conversely, ATRA released from ferroptotic tumor cells activated a RARα–CD38–TFEB–autophagy pathway, increasing MHC-II-dependent antigen presentation by tumor-infiltrating macrophages (208). Macrophages can also protect residual tumor cells through the following mechanisms: macrophage-derived EVs transferred peroxiredoxin 6 (PRDX6), increased intracellular reduced glutathione levels, suppressed lipid peroxidation and mitigated ferroptosis caused by GPX4 inhibition (257). Therefore, RCD crosstalk reshapes macrophage sensing and clearance, while macrophage adaptation can either reinforce ferroptotic tumor control or protect surviving cancer cells.

**Figure 3:**
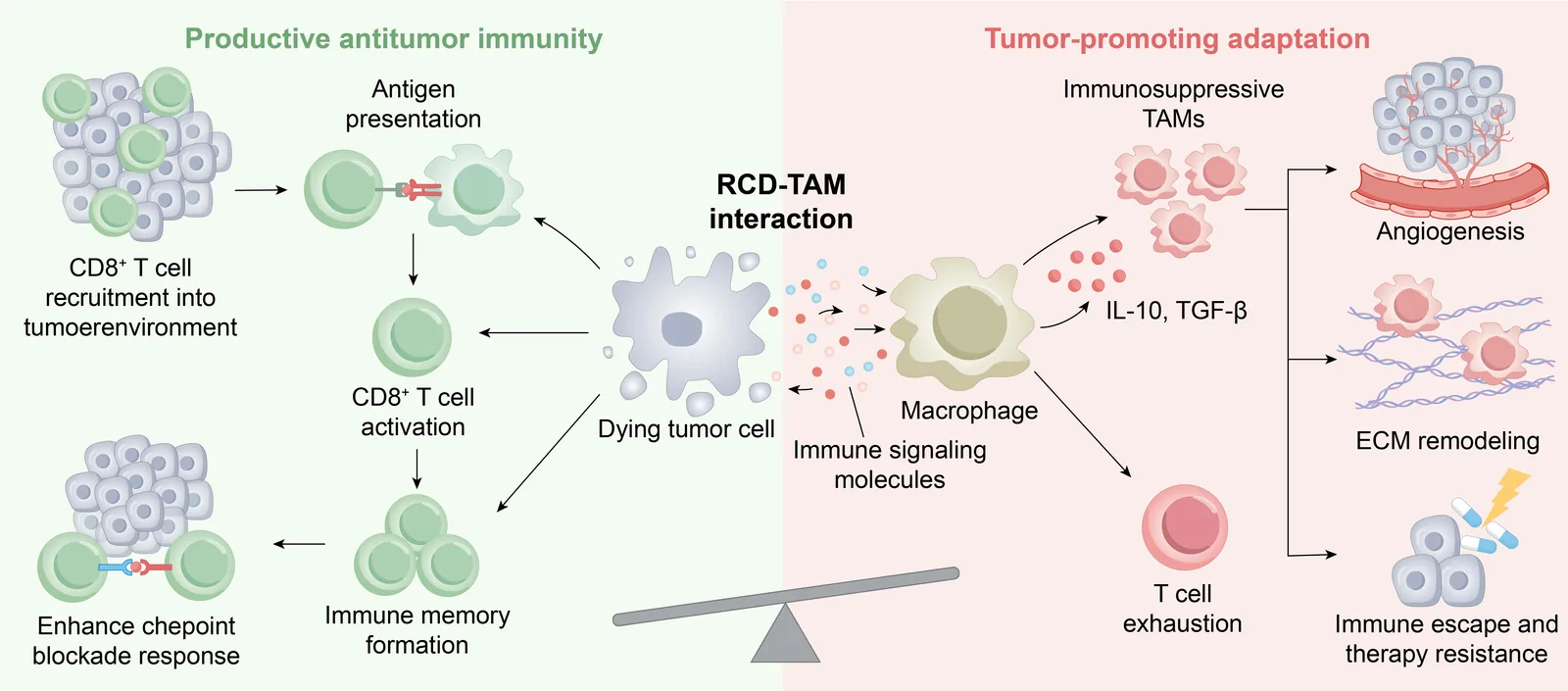
Context-dependent consequences of RCD–macrophage crosstalk for tumor immunity and progression. RCD–macrophage interactions can generate opposing biological outcomes depending on the characteristics of the dying cell, the nature of released signals and the functional state of recipient macrophages. In an immunostimulatory context, RCD-derived signals promote macrophage activation, antigen processing, T-cell recruitment and enhanced responses to immune checkpoint blockade. Conversely, tumour cells can exploit RCD-associated signalling to establish immunosuppressive macrophage programmes, promote angiogenesis, facilitate epithelial–mesenchymal transition and enhance therapeutic resistance. Importantly, inflammatory signalling intensity alone does not predict effective antitumour immunity. Productive immune activation requires coordinated regulation of signal accessibility, macrophage functional state and adaptive immune engagement. Thus, RCD represents a context-dependent communication platform rather than an intrinsically beneficial or detrimental tumor-associated process.

These findings support the use of multi-RCD therapy but also define its limitations. In GSDME-expressing tumor models, irradiation induced pyroptosis and increased cytotoxic T lymphocyte infiltration and tumor suppression (248). Efferocytosis inhibition similarly redirected therapy-induced apoptosis toward secondary necrosis and enhanced the effects of cisplatin, oxaliplatin and radiotherapy in preclinical models (249). A tumor-selective nanoredox lever also potentiated ferroptosis-associated immune stimulation, increased macrophage antigen presentation and enhanced anti-PD-1 therapy (208). However, nonselective phospholipid peroxidation can disable macrophage clearance and promote ferroptosis resistance, whereas NINJ1 blockade can reduce terminal rupture, DAMP release and tissue injury (200, 250). Together, these findings suggest that multi-RCD combinations should coordinate tumor-selective death induction with the preservation of macrophage clearance and timely resolution of inflammatory signals (**Figure 3**).

## 5 Long-range signaling and tumor ecosystem dynamics

The preceding sections focus on local RCD–macrophage interactions within the TME. However, the biological consequences of this axis vary across spatial niches and disease or treatment states (258–260). EVs can extend death-associated signaling beyond the source cell (261), whereas gut- and tumor-associated microbiota reshape the metabolic and immune context in which these signals are generated and interpreted (262, 263). Together, these factors establish a dynamic, context-dependent RCD–macrophage network.

### 5.1 Extracellular vesicle signaling and premetastatic niches

The communication associated with RCD extends beyond soluble DAMPs released in the immediate vicinity of dying cells. EVs enclose proteins, nucleic acids, lipids and metabolites within a lipid bilayer, protecting vulnerable cargo from enzymatic degradation and enabling its transport through biofluids and across biological barriers (264). The apoptosis induced by paclitaxel in triple-negative breast cancer cells increased the release of EVs enriched in CXCL1. These EVs were internalized by macrophages and promoted polarization toward an M2-like phenotype through a mechanism dependent on PD-L1 (265). Macrophages provide a reciprocal source of vesicular signals because their EVs can deliver functional transcripts and proteins that modify the susceptibility of tumor cells to ferroptosis (266, 267). Together, these findings support EVs as regulated carriers that connect the death of tumor cells following treatment with macrophage responses and enable macrophages to influence the fate of residual tumor cells.

EV cargo can alter susceptibility to RCD through the regulation of metal handling and lipid redox homeostasis, as well as through DAMP delivery. EVs derived from TAMs transferred ceruloplasmin messenger RNA (mRNA) to fibrosarcoma cells. Translation of this mRNA enhanced iron export, reduced lipid peroxidation and limited ferroptosis induced by RAS selective lethal 3 (RSL3) (268). In a murine glioblastoma model, TAM-derived TNF-α stimulated tumor cells to release H-ferritin-bound iron through CD63-positive EVs, thereby decreasing intracellular iron availability and reducing sensitivity to RSL3-induced ferroptosis (269). EVs also convey information related to lipid redox homeostasis. A study conducted outside the tumor setting detected increased levels of oxidized polyunsaturated fatty acids and phosphatidylethanolamine species in plasma exosomes from patients with Stevens–Johnson syndrome or toxic epidermal necrolysis (SJS/TEN). However, functional experiments attributed ferroptosis in keratinocytes to exosomal microRNA-375-3p (miR-375-3p), which suppressed ferritin heavy chain 1 (FTH1) and FSP1 without establishing the oxidized lipids as the causal cargo (270). In cancer models, EVs released by macrophages and enriched in PRDX6 increased the abundance of reduced glutathione and suppressed lipid peroxidation, thereby attenuating ferroptosis induced through GPX4 inhibition (257). DAMP cargo can likewise retain biological activity after vesicular transfer: In LPS-induced acute liver injury, macrophage-derived EVs delivered HMGB1 to hepatocytes and triggered RAGE–NLRP3-dependent pyroptosis (271).

Evidence that EVs can propagate cell death and promote the formation of premetastatic niches is currently derived from complementary experimental systems. Pyroptotic cells released EVs carrying assembled GSDMD pores. These pores inserted into the membranes of bystander cells and propagated lytic death both *in vitro* and *in vivo*. This study establishes the extracellular transmission of death signals between cells, but it does not demonstrate the induction of cell death in remote organs in a cancer setting (272). In premetastatic models, exosomes released by lung tumors activated TLR2 and NF-κB signaling in macrophages and induced glycolytic reprogramming. These changes generated immunosuppressive macrophages expressing PD-L1 before overt metastatic colonization (273). In colorectal cancer, EVs enriched in circular RNA-0034880 (circ-0034880) reached the liver through the bloodstream and were taken up by hepatic macrophages. They induced an SPP1^high^CD206^+^ macrophage population that remodeled the stromal environment to support metastasis. Reducing the biogenesis of circ-0034880 attenuated both niche formation and liver metastasis (274). Together, these studies establish an axis in which EVs released by tumors regulate macrophages at remote sites and promote the formation of premetastatic niches. However, the vesicles responsible for niche formation were not traced to a defined RCD program.

Therefore, EV signaling involves the formation of a bidirectional regulatory circuit. RCD in tumor cells or stress caused by treatment can reshape vesicular output, whereas EVs released by macrophages can reset the threshold for cell death in surviving tumor cells. Depending on their cargo and recipient context, EVs may propagate death between cells or reinforce resistance to ferroptosis, immunosuppression and metastatic conditioning. However, the studies considered here do not yet connect a defined RCD program in tumor cells, the selective loading of EV cargo, cell death at remote sites and the formation of premetastatic niches mediated by macrophages within a single experimental model. Tracing EVs according to their cellular lineage, mapping their uptake in specific organs and selectively blocking individual cargo molecules will be necessary to establish this causal sequence.

### 5.2 Microbiota regulation of cell death and macrophage function

Gut and intratumoral microbes reshape the TME through metabolites, microbial products and host cell interactions (275–277). In esophageal squamous cell carcinoma, *Fusobacterium* nucleatum induced macrophage mitophagy, reduced mitochondrial ROS levels and supported bacterial persistence; infected macrophages promoted metastasis through CCL2–CCR2 signaling (278). In gastric cancer, vesicles from intratumoral Ligilactobacillus salivarius delivered 2,3-bisphosphoglycerate-dependent phosphoglycerate mutase and activated formyl peptide receptor 1 (FPR1), MAPK and NF-κB signaling, enhancing macrophage inflammatory activity and the response to PD-1 blockade (279). These studies define microbial routes that alter macrophage mitochondrial fitness and the inflammatory state. Neither ferroptosis nor cuproptosis was established as the underlying mechanism.

Ferroptosis provides the clearest mechanistic link between microbial metabolites and cell fate in the TME. Butyrate inhibited class I histone deacetylases (HDACs), induced cellular Fos proto-oncogene (c-Fos) and suppressed SLC7A11, thereby reducing glutathione synthesis and sensitizing colorectal cancer cells to ferroptosis (280). In contrast, trans-3-indoleacrylic acid from Peptostreptococcus anaerobius activated aryl hydrocarbon receptor (AHR) and aldehyde dehydrogenase 1 family member A3 (ALDH1A3), increased the availability of reduced nicotinamide adenine dinucleotide (NADH) to the FSP1–CoQ10 antioxidant system and protected colorectal cancer cells (281). In bladder cancer, indole-3-pyruvic acid maintained macrophage redox homeostasis through AHR, NF-κB and SLC7A11, limited macrophage ferroptosis and preserved CD8^+^ T-cell activity (282). Thus, metabolite identity, recipient cells and antioxidant pathways determine ferroptotic susceptibility. Macrophage-targeted liposomal delivery produced the strongest therapeutic effects, and whether microbiota-induced tumor cell ferroptosis subsequently reprograms macrophages remains unresolved.

Evidence connecting the microbiota to cuproptosis in the TME remains fragmented. In macrophages differentiated from the human monocytic THP-1 cell line, LPS induced TLR4-dependent hexokinase domain containing 1 (HKDC1) expression and glycolysis, reduced cuproptosis and increased inflammatory activity; HKDC1 depletion restored copper-dependent death in a sepsis model (283). In breast cancer, butyrate suppressed TLR4 expression and increased pyridoxal kinase (PDXK) and solute carrier family 25 member 28 (SLC25A28) expression, but canonical copper-dependent death and macrophage responses were not examined (284). Material released from elesclomol-treated colorectal cancer cells reduced galactose-3-O-sulfotransferase 4 (GAL3ST4) and CD163 expression in M2-like macrophages and weakened their tumor-promoting activity (285, 286). These studies link microbial signals, cuproptosis-associated changes and macrophage state only in separate models. Direct evidence that a defined intestinal or intratumoral microbial metabolite induces canonical cuproptosis and thereby reprograms TAMs is still lacking.

### 5.3 Spatial and temporal dynamics

RCD and macrophage interactions vary with anatomical site and treatment history. Their effects depend on whether tumor cells remain viable or complete death, which signals persist and which macrophage populations process them (260, 287). Therefore, primary tumors, invasive fronts, metastatic lesions and posttreatment lesions represent distinct, but not necessarily sequential, regulatory states.

#### 5.3.1 Primary tumors, invasive fronts and metastases

In primary tumor models, macrophages regulate both death execution and the response to completed apoptosis. Phosphatidylserine exposed by apoptotic tumor cells activated phosphatidylserine receptor, STAT3 and JMJD3 signaling in macrophages, inducing immunosuppressive gene expression and promoting tumor growth (98). Under hypoxia, TAMs transferred ceruloplasmin mRNA to fibrosarcoma cells through EVs, lowering intracellular iron and lipid peroxidation and limiting ferroptosis induced by RSL3 (268). At the invasive front, slow-cycling mesenchymal stromal cells expressing platelet-derived growth factor receptor alpha (PDGFRα) and disintegrin and metalloproteinase 12 (ADAM12) accumulated at tumor margins and promoted macrophage efferocytosis, immunosuppressive reprogramming and pathological angiogenesis through Gas6, Lgals3 and Csf1. Their depletion reduced hypoxia and acidosis, increased effector T-cell infiltration and restricted tumor growth in responsive models (288). At metastatic sites, macrophages can also process death signals derived from injured host tissues rather than tumor cells alone. During liver colonization by PDAC, metastasis-associated macrophages (MAMs) cleared dead hepatic parenchymal cells. Macrophage-derived progranulin (PGRN) promoted cystic fibrosis transmembrane conductance regulator (CFTR)-dependent lysosomal acidification and corpse degradation, activating liver X receptor alpha (LXRα) and retinoid X receptor alpha (RXRα), inducing ARG1 expression and suppressing CD8^+^ T-cell function. The inhibition of efferocytosis or macrophage PGRN expression reduced metastatic outgrowth (289). A separate study revealed that MLKL-dependent necroptosis in pancreatic tumors increased macrophage recruitment, tumor cell CD47 expression and macrophage extracellular trap formation. These traps activated CXCL8 signaling, promoted epithelial-to-mesenchymal transition and facilitated liver metastasis, whereas combined inhibition of necroptosis and CD47 restricted dissemination (122). These findings indicate that the axis shifts from the regulation of tumor cell death within primary lesions to stromally organized clearance at invasive margins and organ-conditioned immune suppression at metastatic sites. Whether nonapoptotic RCD modalities are selectively enriched or differentially processed at these spatial boundaries remains unclear.

#### 5.3.2 Treatment, residual disease and recurrence

**Figure 4:**
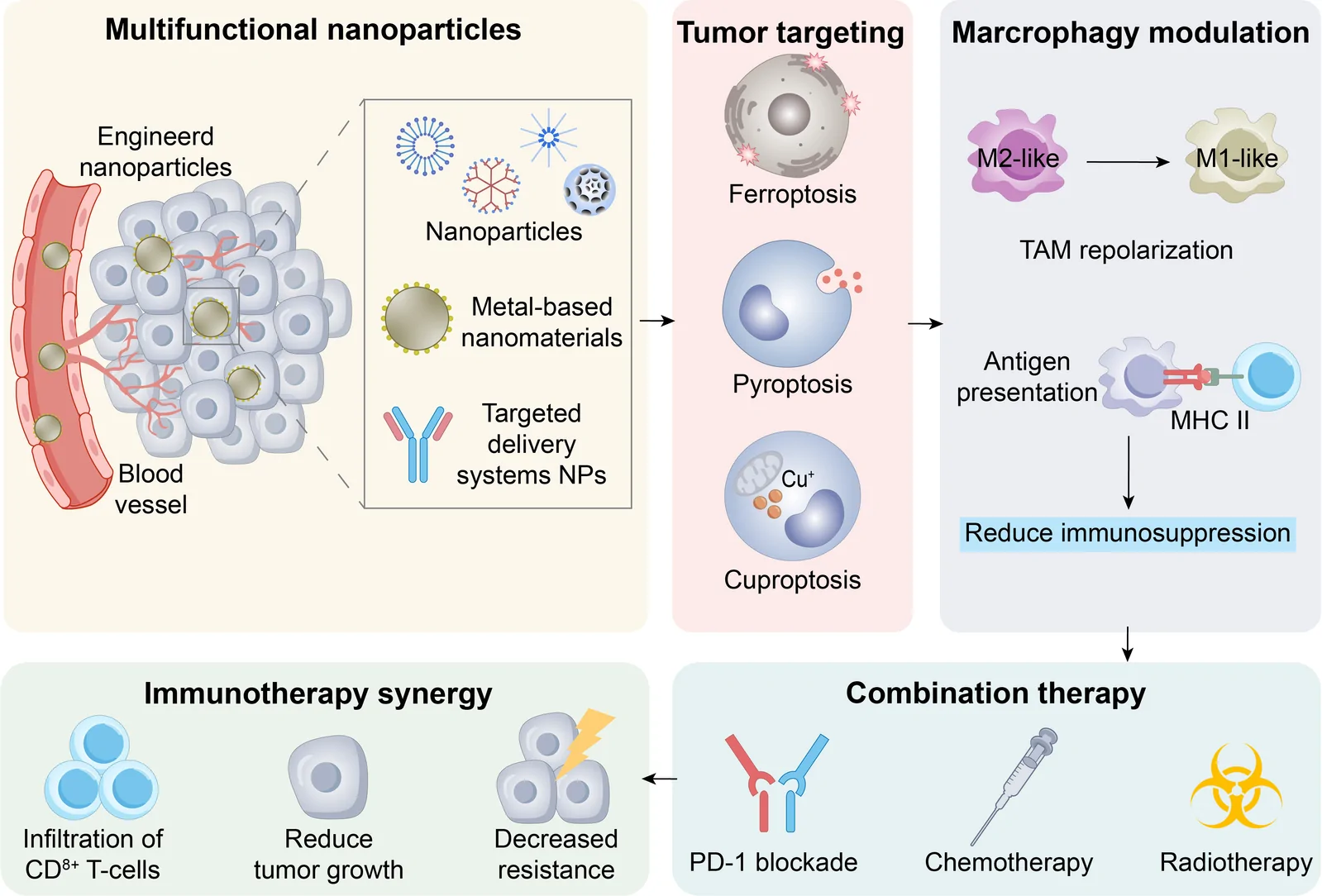
Therapeutic strategies targeting the RCD–macrophage axis for precision cancer immunotherapy. Targeting the RCD–macrophage axis provides opportunities to remodel the tumor immune microenvironment and enhance therapeutic responses. Emerging strategies include selective induction of immunogenic RCD modalities, development of tumor-targeted nanoplatforms, modulation of macrophage metabolic states and combination approaches integrating RCD-based therapies with immune checkpoint blockade, chemotherapy or radiotherapy. Effective therapeutic design requires precise control of the targeted cell population, preservation of macrophage functions involved in antigen processing and clearance, and consideration of tumor-specific metabolic and spatial contexts. Future approaches will likely depend on patient stratification strategies that identify the dominant RCD pathway, macrophage state and immune vulnerability within individual tumors.

Treatment produces a rapid increase in dying-cell material such that macrophages can redirect toward tumor persistence. In TNBC, paclitaxel-induced apoptosis resulted in the release of CXCL1-enriched EVs that activated PD-L1-associated immunosuppressive signaling in macrophages and promoted chemoresistance, invasion and metastasis (290). In ovarian cancer, chemotherapy increased the number of macrophages containing apoptotic tumor cell material. Efferocytosis induced ornithine decarboxylase 1 (ODC1)-dependent polyamine metabolism, increased macrophage osteopontin (OPN) levels and promoted tumor cell stemness through CD44. The inhibition of efferocytosis or ODC1 expression restored cisplatin sensitivity and limited tumor regrowth (291). These programs become spatially organized in residual and treatment-resistant lesions. HCC samples collected after chemoembolization contained neighborhoods of PD-L1^+^ macrophages, stem-like residual tumor cells and exhausted CD8^+^ T cells. Macrophage-derived TGF-β1 supported residual cell persistence, whereas combined inhibition of PD-L1 and TGF-β eliminated residual stem-like cells in mouse models (292). In serial biopsies from patients with HCC resistant to immune checkpoint blockade, TREM2^+^ lipid-associated macrophages (LAMs) transferred fatty acids acquired through efferocytosis back to tumor cells in EVs, supporting energy production and activating the MYC and TGF-β programs. TREM2 disruption restored treatment sensitivity (293). At clinical recurrence, multiomic analysis of matched primary and recurrent glioblastomas (GBMs) revealed changes in malignant cell states, nonmalignant cell composition and tumor-immune communication but did not identify the responsible death signals (294). Current evidence supports a staged model in which treatment-induced RCD is followed by macrophage reprogramming, residual cell survival and recurrent ecosystem remodeling. It does not establish persistence of the same RCD signal or macrophage lineage across these states. Longitudinal studies combining modality-specific RCD reporters, macrophage lineage tracing and spatial multiomics are needed to elucidate this relationship (**Figure 4**).

## 6 Nanomedicine strategies and clinical translation

Nanomedicine can deliver RCD-inducing agents to tumor cells and alter how macrophages respond to dying cells. This dual action is important because macrophages can remove dying cells, present tumor antigens or suppress antitumor immunity (295). However, greater material complexity does not necessarily improve therapeutic value. A useful platform must induce RCD mainly in tumor cells, produce a defined macrophage response and avoid uncontrolled inflammation. Most systems designed for this purpose remain at the preclinical stage (296).

### 6.1 Nanomedicine design and cellular targeting

Nanoplatforms act at three main points along the RCD–macrophage axis. Some deliver RCD inducers to tumor cells and rely on the resulting DAMPs, metabolites or cellular debris to activate macrophages. For example, a tumor microenvironment-responsive calcium-based nanoinducer triggered GSDME-dependent pyroptosis in tumor cells and the release of DAMPs. The oxygen generated during nanoparticle degradation and inflammatory signaling further promoted M1-like TAM polarization, dendritic cell maturation and CD8^+^ T-cell infiltration (297). Others directly alter macrophage phagocytosis, metabolism or inflammatory signaling. For example, lanthanum nickel oxide nanozymes hydrolyzed intracellular ATP and promoted protein dephosphorylation in TAMs, thereby activating AMP-activated protein kinase (AMPK)–mTOR signaling, inducing autophagy and reprogramming M2-like TAMs toward an M1-like state (298). A third group combines both functions. For example, a metal–organic framework nanovaccine induced pyroptosis and cuproptosis in tumor cells while releasing an extracellular signal-regulated kinase 5 inhibitor that reduced macrophage efferocytosis. This delayed corpse removal, increased the release of tumor-derived material and enhanced antitumor immunity (299). In another model, a hypoxia-responsive nanoplatform selectively increased ferroptosis in tumor cells. ATRA released from these cells activated RARα, CD38 and TFEB signaling in macrophages, which increased MHC-II-dependent antigen presentation and improved the response to anti-PD-1 treatment (208). These studies show that nanoplatforms can link tumor cell death to a defined macrophage function. However, they do not yet establish clinical efficacy. Therapeutic design should account for the RCD pathway, the magnitude of tumor cell killing and its temporal profile (300). These features can alter the kinetics of lethal execution and the immunogenic properties of dying tumor cells, making endpoint killing an incomplete measure of therapeutic effect (301). Preclinical evaluation should integrate time-resolved measurements of tumor cell death with direct assessments of macrophage function and tumor response (302).

Effective delivery remains a major challenge. Nanoplatforms may use tumor accumulation, receptor-binding ligands, microenvironment-responsive release, external energy or biomimetic carriers. Yet, accumulation within a tumor does not prove selective entry into tumor cells. Similarly, uptake by macrophages does not prove selective targeting of immunosuppressive TAM subsets. A meta-analysis of 273 preclinical tumor studies revealed that compared with single-drug treatments, multidrug nanotherapies generally resulted in stronger tumor control and that coencapsulation was more effective than single-drug delivery was (303). These findings support combined delivery, but they remain based mainly on animal models. Future studies should identify which cells take up the platform, confirm that the intended RCD pathway is required for efficacy and measure macrophage functions such as phagocytosis or antigen presentation. Changes in tumor fluorescence or M1 and M2 markers alone are insufficient.

### 6.2 Clinical feasibility, safety and patient selection

The clinical evidence differs greatly among RCD pathways. Apoptosis is the most clinically established target. The B-cell lymphoma 2 inhibitor venetoclax has shown that restoring apoptosis can substantially benefit selected hematological malignancies, although activity in solid tumors remains limited and depends on tumor-specific apoptosis (304). Autophagy inhibitors have also entered clinical trials, but the results have been inconsistent. In a randomized phase II study of platinum-sensitive relapsed ovarian cancer, the addition of hydroxychloroquine to chemotherapy did not improve response or survival (305). In contrast, nanotherapies specifically designed to induce pyroptosis, necroptosis, ferroptosis or cuproptosis remain largely preclinical. Some approved drugs can affect these pathways, but this does not prove that a specific RCD program explains their clinical activity. Current cancer nanomedicines have succeeded mainly by improving drug exposure and reducing systemic toxicity rather than by achieving precise cell-specific targeting (306).

Safety depends on both the carrier and the induced RCD pathway. Nanoparticles can activate complement, bind plasma proteins and accumulate in phagocyte-rich organs, which may contribute to infusion reactions and altered clearance (307). The death pathway can add further risk. Pyroptosis and necroptosis release inflammatory mediators; thus, broad activation may damage normal tissues or result in systemic cytokine release. The clinical frequency of this complication in RCD-inducing nanotherapies is not yet known (308, 309). Ferroptosis can also injure normal tissues through uncontrolled lipid peroxidation, and many experimental ferroptosis inducers have unsuitable pharmacokinetic properties (310, 311). Copper-based systems must limit copper exposure outside the tumor because both copper excess and copper deficiency are harmful (312). Complex formulations create additional problems in manufacturing. The particle size, drug loading, release rate and stability must remain consistent during large-scale production. These issues favor simpler platforms with tumor-restricted activation and clearly measurable safety markers.

Patient selection should answer three practical questions. First, can the platform reach the tumor? A histopathological score based on tumor blood vessel density and TAM abundance predicted the accumulation of liposomal doxorubicin in mouse models and human tumor specimens, suggesting that tissue structure may help identify patients with favorable drug delivery (313). Second, can the selected RCD pathway still operate in the tumor? Related execution proteins and resistance mechanisms should be assessed with functional assays rather than gene expression alone. Third, can the immune system respond after tumor cell death? Tumors require retained antigen-presentation capacity, functional or recoverable T cells and macrophages that can still be reprogrammed. In TNBC, combining baseline and early treatment spatial features improved the prediction of the immune checkpoint blockade response (314). In clear-cell renal cell carcinoma, high infiltration by SPP1-positive TAMs was associated with poor immunotherapy response and impaired CD8-positive T-cell function (315). Therefore, patients are most likely to benefit when the nanoplatform reaches the tumor, the intended RCD pathway remains functional, and the local immune response can still be restored.

## 7 Conclusions and perspectives

The evidence reviewed here supports a framework in which the consequences of RCD are determined not by the death modality alone but by how its signals are generated, accessed and interpreted within the tumor microenvironment. During early execution, apoptotic cells generally retain their plasma membrane integrity and present macrophages with surface clearance cues, selected soluble mediators and membrane-enclosed cargo. Necroptosis and pyroptosis result in earlier membrane permeabilization and broadened access to intracellular material, although cytokine secretion, pathway activation and terminal rupture do not necessarily occur together. Ferroptosis generates a lipid-centered signaling profile that changes with the stage of phospholipid peroxidation, whereas the extracellular signals specific to cuproptosis remain less well defined. Autophagy-related processes differ from these execution programs because they often regulate cellular adaptation, signal production and cargo processing without directly causing death; therefore, autophagy-dependent cell death should be assigned only when the autophagic machinery is required for lethality. These differences shape what macrophages can recognize, internalize and process. The outcome further depends on the identity and death stage of the source cell, the kinetics and magnitude of cell death, and the composition and persistence of the signal package. It also depends on the origin and functional state of the recipient macrophage and on the spatial and therapeutic context. Macrophages also act in the reverse direction by altering tumor cell death thresholds, although the strength and nature of this evidence differ among RCD programs. Therefore, the RCD–macrophage axis is a reciprocal system in which death execution, corpse processing and macrophage feedback jointly determine tumor control, immune suppression and treatment resistance.

The first priority is to define the causal sequence linking each of the six RCD modalities considered here—apoptosis, necroptosis, pyroptosis, ferroptosis, cuproptosis and autophagy-related regulation, including autophagy-dependent cell death where causally established—to macrophage function. Current studies often equate pathway activation or RCD-related transcriptional signatures with completed cell death and infer macrophage activity from changes in M1- or M2-associated markers. These approaches do not establish whether irreversible death has occurred, which signals have become accessible or which macrophage function has changed. They also do not define how death kinetics or lethal fraction relate to macrophage responses. Future studies should verify the death modality in a defined cell population and quantify death onset, death rate and lethal fraction over time. They should then identify the relevant surface cues, soluble mediators or cellular cargo, perturb their recognition or intracellular processing in a specified macrophage population, and measure corpse clearance, antigen presentation, cytokine production, metabolic adaptation and lymphocyte regulation. The same level of resolution is required to analyze the crosstalk among the six RCD modalities. Apoptosis, necroptosis and pyroptosis can be redirected through caspase-8 activity, gasdermin cleavage and failed corpse clearance. Autophagy can alter stress tolerance, intracellular cargo processing and susceptibility to apoptosis, necroptosis, pyroptosis and ferroptosis; however, its mechanistic relationship with cuproptosis remains unclear, and autophagic activity should not be equated with autophagy-dependent cell death. Ferroptosis and cuproptosis are linked to distinct but intersecting redox, metal and mitochondrial metabolic constraints; current evidence supports pharmacological cooperation but not a confirmed endogenous switch between these modalities. Time-resolved comparisons within the same tumor model are needed to distinguish genuine pathway conversion from parallel activation or compensatory signaling and to determine how these interactions change the signal package ultimately processed by macrophages.

A second priority is to define how macrophage heterogeneity and spatiotemporal context alter the decoding of RCD-derived signals. Tissue-resident and monocyte-derived macrophages differ in developmental history, tissue adaptation and access to tumor niches, whereas SPP1-, C1QC-, TREM2- and LYVE1-enriched programs may overlap and do not confer fixed functions across cancers. Future studies should identify which macrophage populations encounter dying cells, which receptors mediate signal recognition, how engulfed cargo is processed and whether the resulting function is inflammatory, antigen-presenting, tissue-remodeling or immunoregulatory. Lineage tracing, spatial multiomics and serial sampling will be needed to follow these interactions from the primary tumor and invasive front to metastatic lesions, treatment-induced residual disease and recurrence. Such approaches should determine whether treatment preserves the same macrophage population and signal-decoding program or replaces them with new cellular states. Local interactions must also be connected to long-range regulation. EVs can transport proteins, nucleic acids, metal-regulatory components, antioxidant enzymes and lipid-related cargo; however, no single model has yet established a complete causal sequence from a defined RCD program to selective EV loading, uptake by macrophages at a distant site and formation of a premetastatic niche. The microbiota–TME axis remains similarly fragmented. Microbial metabolites can alter tumor cell ferroptotic susceptibility or the macrophage redox state, but direct evidence that a defined intestinal or intratumoral metabolite induces canonical ferroptosis or cuproptosis and subsequently reprograms TAM function remains limited. Emerging or proposed death programs, including disulfidptosis, ammonoptosis, mitoxyperiosis and floatptosis, may expand this network, but their relevance to cancer should be evaluated using the same criteria applied to established RCD modalities: irreversible execution, defined signal output, recognition by a specified macrophage population and a causal effect on tumor biology (316–319).

Clinical translation will require these mechanistic principles to be converted into selective, measurable and feasible interventions. Among apoptosis, necroptosis, pyroptosis, ferroptosis, cuproptosis and autophagy-related regulation, apoptosis has the clearest clinical precedent, whereas clinical studies of autophagy modulation have produced inconsistent results. Therapeutic strategies centered on necroptosis, pyroptosis, ferroptosis or cuproptosis, particularly those designed to coordinate tumor cell death with macrophage regulation, remain predominantly preclinical (305, 319). Nanoplatforms provide opportunities to coordinate tumor cell death with macrophage regulation, but increasing material complexity does not ensure biological selectivity or clinical value. A platform must reach the intended tumor compartment, enter the relevant target cell, engage a functional RCD pathway and generate the macrophage response required for therapeutic benefit. Tumor accumulation alone does not demonstrate tumor cell selectivity, and macrophage uptake does not establish targeting of a specific TAM population. Therefore, multi-RCD combinations should be designed around the death competence of the tumor, the cellular source of each signal, the intended macrophage function and the sequence in which death programs are induced. Safety is equally important. Broad activation of pyroptosis or necroptosis may cause normal-tissue injury and systemic inflammation; ferroptosis may damage nonmalignant tissues through lipid peroxidation, and copper-based systems must control systemic metal exposure. Manufacturing reproducibility and formulation stability further favor simpler platforms with tumor-restricted activation and defined pharmacodynamic markers. Patient selection should integrate three conditions: whether the therapy can reach the tumor, whether the intended RCD pathway remains executable and whether local macrophage, antigen-presentation and T-cell functions remain recoverable. The aim should not be to maximize tumor cell death in isolation but to control which cells die, the kinetics and magnitude of death, which signals remain available and how defined macrophage populations process those signals.
